# Design and Synthesis of Piperazine-Based Compounds Conjugated to Humanized Ferritin as Delivery System of siRNA in Cancer Cells

**DOI:** 10.1021/acs.bioconjchem.1c00137

**Published:** 2021-05-12

**Authors:** Natalia Pediconi, Francesca Ghirga, Cristina Del Plato, Giovanna Peruzzi, Constantinos M. Athanassopoulos, Mattia Mori, Maria Elisa Crestoni, Davide Corinti, Franco Ugozzoli, Chiara Massera, Alessandro Arcovito, Bruno Botta, Alberto Boffi, Deborah Quaglio, Paola Baiocco

**Affiliations:** † Center for Life Nano- & Neuro-Science, Fondazione Istituto Italiano di Tecnologia (IIT), V.le Regina Elena 291, 00161 Rome, Italy; ‡ Department of Chemistry and Technology of Drugs, “Department of Excellence 2018−2022”, Sapienza University of Rome, P.le Aldo Moro 5, 00185 Rome, Italy; § Department of Chemistry, University of Patras, GR-26504 Rio-Patras, Greece; ∥ Department of Biochemical Sciences “Alessandro Rossi Fanelli”, Sapienza University of Rome, P.le A. Moro 5, 00185 Rome, Italy; ⊥ Department of Biotechnology, Chemistry and Pharmacy, “Department of Excellence 2018−2022”, University of Siena, via Aldo Moro 2, 53100, Siena, Italy; # Department of Engineering and Architecture, University of Parma, Parco Area delle Scienze 181/A, 43124 Parma, Italy; ¶ Department of Chemical Sciences, Life and Environmental Sustainability, University of Parma, Parco Area delle Scienze 17/A, 43124 Parma, Italy; □ Dipartimento di Scienze Biotecnologiche di base, Cliniche Intensivologiche e Perioperatorie, Università Cattolica del Sacro Cuore, Largo F. Vito 1, 00168, Roma, Italy; ● Institute of Molecular Biology and Pathology, National Research Council, P.le A. Moro 7, 00185 Rome, Italy

## Abstract

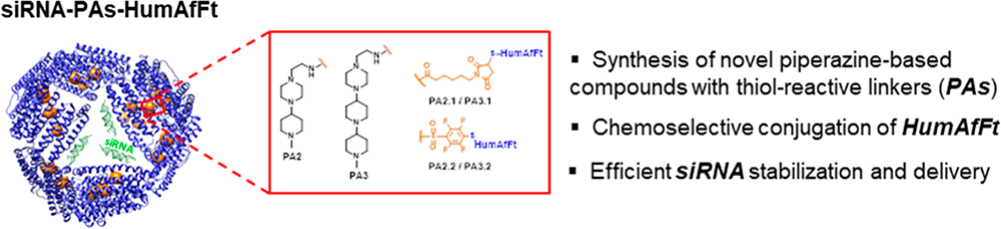

Gene expression regulation by small interfering RNA (siRNA) holds promise in treating a wide range of diseases through selective gene silencing. However, successful clinical application of nucleic acid-based therapy requires novel delivery options. Herein, to achieve efficient delivery of negatively charged siRNA duplexes, the internal cavity of “humanized” chimeric Archaeal ferritin (HumAfFt) was specifically decorated with novel cationic piperazine-based compounds (PAs). By coupling these rigid-rod-like amines with thiol-reactive reagents, chemoselective conjugation was efficiently afforded on topologically selected cysteine residues properly located inside HumAfFt. The capability of PAs-HumAfFt to host and deliver siRNA molecules through human transferrin receptor (TfR1), overexpressed in many cancer cells, was explored. These systems allowed siRNA delivery into HeLa, HepG2, and MCF-7 cancer cells with improved silencing effect on glyceraldehyde-3-phosphate dehydrogenase (GAPDH) gene expression with respect to traditional transfection methodologies and provided a promising TfR1-targeting system for multifunctional siRNA delivery to therapeutic applications.

## Introduction

**Figure 1:**
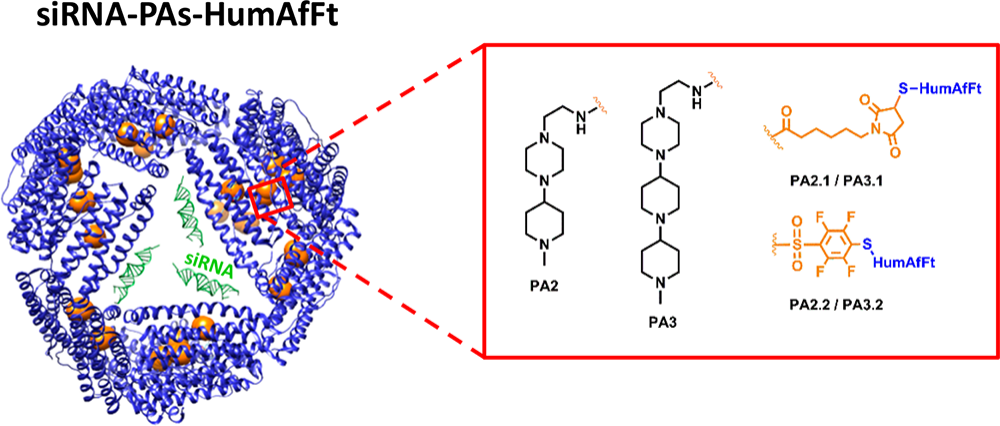
Schematic presentation of **siRNA-PAs-HumAfFt** delivery systems. As depicted in the red box, the orange spheres inside HumAfFt cavity (blue cartoon) represent piperazine-based compounds featuring one (**PA2**) or two piperidine rings (**PA3**) attached through thiol-reactive groups (**PA2.1**/**PA3.1** or **PA2.2**/**PA3.2**) to topologically selected protein cysteine residues. The siRNA molecules are depicted as green duplexes.

Small interfering RNA (siRNA) represents a revolutionary tool for gene therapy with a wide array of potential applications in the regulation of gene expression. However, successful employment of nucleic acid-based therapy still suffers from limitations because of the extremely labile nature of siRNA under physiological conditions, which hamper its efficient and sustained delivery. RNA molecules are indeed susceptible to chemical degradation due to the presence of intense extracellular nuclease activities and scavenging activity by the immune system.^1−3^ In the last years, many nucleic acid delivery vectors including cationic lipids and polymers have been explored to circumvent these restrictions and to reach the best compromise between transfection efficiency and cytotoxicity.^4,5^ Nanoparticle-based delivery systems have been widely used for their ability to protect the siRNA cargo from nuclease activity, for tissue targeting and cell specificity, and for efficient cell membrane crossing properties.^6^ However, there are significant concerns regarding their safety and biocompatibility when used for human therapy. In addition, the procedures to prepare these vehicles are laborious and time-consuming. Protein-mediated siRNA delivery has several advantages such as facile chemical modifications and good biocompatibility, which may overcome various hurdles associated with efficient siRNA delivery.^7,8^ Ferritin nanocages emerged as ideal delivery systems endowed with a well-defined hollow spherical architecture with inner and outer diameters of 8 and 12 nm, respectively, and precisely self-assembled from 24 copies of identical 20 kDa subunits. These systems can be easily and accurately manipulated by genetic modifications to enhance their loading cargo properties with appropriate chemical bioconjugations.^9−13^ Ferritin nanocages display homogeneity, low production costs, improved thermal stability, and cellular uptake activity of small bioactive compounds. So far, the surface of human ferritin (hFt) nanoparticles was engineered with multiple peptides (e.g., cationic peptide for siRNA conjugation, cell targeting, and cell-penetrating peptides) to achieve the siRNA transport inside cytoplasm of target cancer cells.^14^ More importantly, ferritin vehicles were able to shield the negative electrostatic charge of siRNA upon encapsulation of the latter^15^ and promote efficient intracellular delivery through the transferrin receptor (TfR1 or CD71) which is overexpressed in many cancer cells in response to the increased demand of iron.^16^ Targeting TfR1, in order to deliver drugs in highly proliferating cancer cells, thus has been confirmed to be an optimal strategy to intervene with the progression of cancer.^17,18^ As highlighted by very recent structural studies,^19^ the external unstructured loop region of hFt is crucial for the complex formation with TfR1. Alongside, an engineered ferritin from hyperthermophile *Archaeoglobus fulgidus* (AfFt), displaying a unique three-dimensional structure with four wide triangular pores on the surface and characterized by unusual salt-triggered assembly disassembly properties which allowed a reversible opening and closing of the nanoparticle at neutral pH, was successfully endowed with the human H homopolymer recognition sequence by TfR1.^20^ The protein was accordingly named “Humanized archaea ferritin” (HumAfFt) and was demonstrated to be easily produced in large amounts and loaded with a wide range of compounds including small proteins.^21−23^ To this aim, HumAfFt has been engineered with a point mutation (M54C) in order to provide a highly reactive thiol group inside the protein shell. This mutation, specifically located in the inner cavity of the protein, introduces 24 novel attachment sites to covalently and selectively link numerous functionalities. Thus far, HumAfFt was successfully used as a template for multifunctional delivery nanoplatforms.^21−23^ Accordingly, HumAfFt appears to be an ideal tool to encapsulate siRNA molecules in the 8-nm-diameter large cavity by increasing nucleic acid stability, and to selectively target malignant cells via TfR1 receptor. However, the protein inner cavity features many negatively charged residues making the possibility to encapsulate siRNA molecules very unlikely. In the present paper, our strategy implies the introduction of positively charged functionalities by using novel cationic polyamines (**PAs**) to physically entrap siRNA duplexes, thus providing efficient cellular uptake and excellent protection of siRNAs against serum or RNase. To this aim, piperazine-based compounds featuring one or two piperidine rings, hereafter named **PA2** and **PA3**, respectively, were rationally designed and synthesized to promote electrostatic interactions with negative small nucleic acids.^24^ In addition, thiol-reactive groups such as maleimide in compounds **PA2.1** and **PA3.1** and pentafluorobenzenesulfonamide in compounds **PA2.2** and **PA3.2**, respectively, were efficiently incorporated in order to ensure selective modification of one cysteine residue per monomer of HumAfFt (Figure 1). Hence, the increase of positive charges inside the HumAfFt promoted the spontaneous supramolecular association with siRNA molecules into the corresponding **siRNA-PAs-HumAfFt** nanoparticles (Figure 1). We further explored the possibility to employ **PAs-HumAfFt** as as safe and effective targeting shuttle of noncovalently loading siRNAs for mediating the downregulation of glyceraldehyde-3-phosphate dehydrogenase (GAPDH) gene expression, a housekeeping gene implicated in the catalysis of an important energy step in carbohydrate metabolism. The efficiency of the siRNA delivery system was evaluated *in vitro* against a variety of malignant human cell lines, including HeLa (cervical adenocarcinoma), MCF-7 (breast cancer), and HepG2 (hepatocellular carcinoma), which are known to be particularly resistant to traditional transfection methods.^15,25^ However, it is envisioned that the reported nanodelivery systems might be employed to multiple siRNA-based silencing for a wide range of biotechnological applications.

## Results and Discussion

### Design and Synthesis of Polyamines to Enhance siRNA Affinity to HumAfFt

**Scheme 1:**
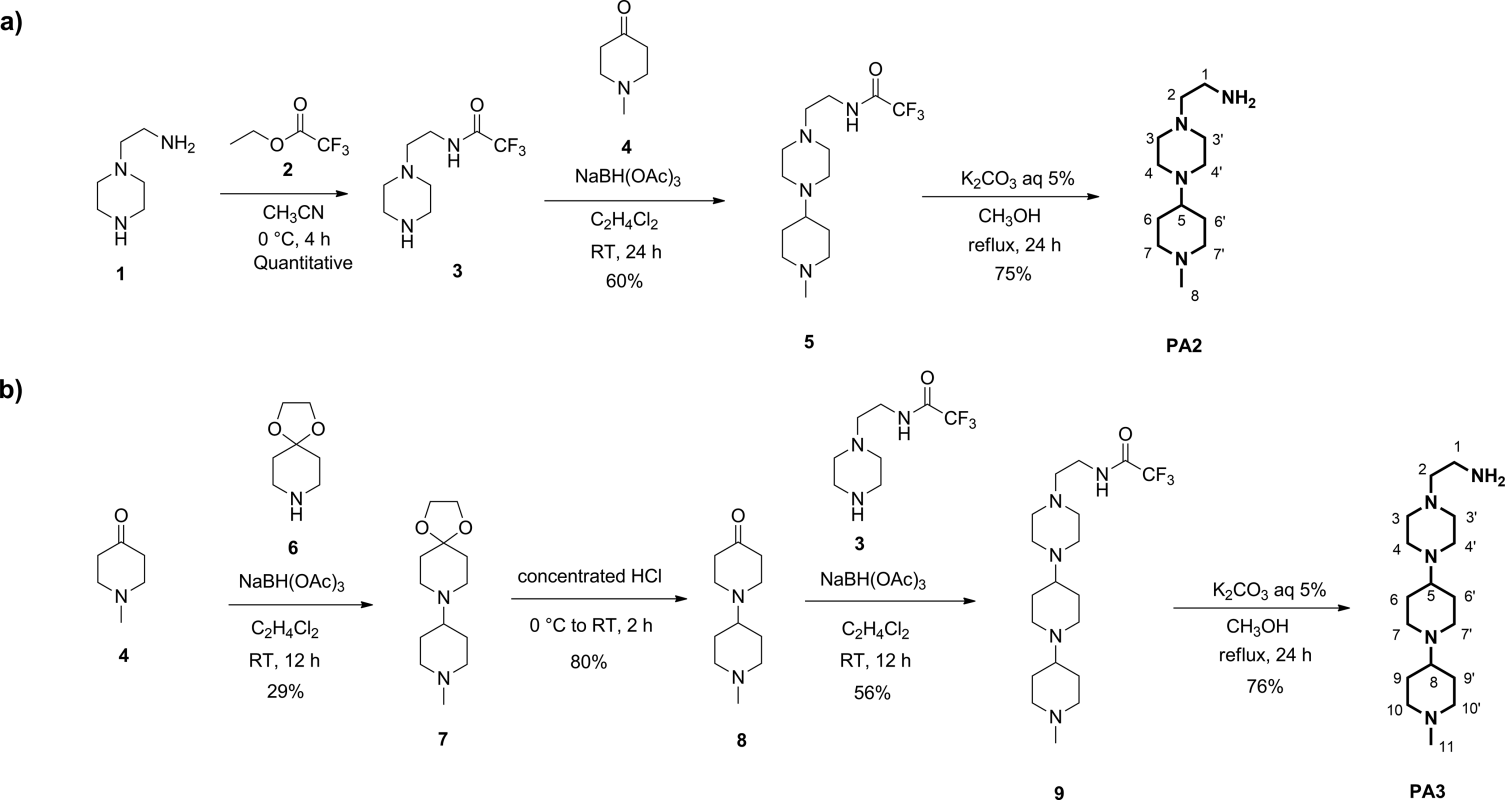
Synthetic Procedures for Polyamines **PA2** (a) and **PA3** (b), Respectively

The vulnerability of nude therapeutic RNAs in biological environments, including their short half-life, low stability, and transfection efficiency *in vivo*, strongly suggest the compelling need for siRNA shelf-life improvement. Encapsulation into HumAfFt nanocages was thus considered as a promising strategy to increase siRNA stability and delivery to target cells. In this scenario, we successfully designed and synthesized novel polyamines bearing sulfhydryl reactive linkers to positively charge the inner cavity of HumAfFt protein, thus promoting the supramolecular interaction with siRNA.^27,28^ As predicted by theoretical p*K*_a_ values calculated through an *ab initio* quantum chemical program (Jaguar), the amine groups may significantly contribute to the electrostatic attraction and incorporation of siRNA molecules into the HumAfFt at a physiological pH (Figure S26). The choice to design cyclic amines despite the well-known linear ones (e.g., spermidine and spermine) lies in laborious and time-consuming synthetic procedures of the latter, requiring several protection/deprotection steps of primary and secondary amines. In this regard, a simple, inexpensive, and widely accessible method was developed for the preparation of rigid-rod-like piperazine-based compounds containing one (**PA2**) or two piperidine (**PA3**) moieties in excellent yields.^29^ As outlined in Scheme 1a,b, the elongation strategy of the 2,3,5,6-tetrahydropyrazine scaffold was based on direct reductive amination.^30^ Since primary amines also undergo this reaction, the preparation of **PA2** started from the selective protection of commercially available 2-(piperazin-1-yl)ethanamine (**1**) by using ethyltrifluoroacetate reagent (**2**) (Scheme 1a).^31^ The reaction afforded quantitatively compound **3**, which was allowed to react with *N*-methyl-4-piperidone (**4**) in a one-pot procedure by treatment with triacetoxyborohydride, a mild and selective reducing agent.^29,30^ The obtained compound **5** (60% yield) was further deprotected under mild basic conditions to give the desired amine **PA2** in good yield (Scheme 1a). Similarly, the synthesis of **PA3** (Scheme 1b) started with a reductive amination reaction between ketone **4** and 1,4-dioxa-8-azaspiro[4.5]decane (**6**), affording the *di*-piperidine compound **7** in 29% yield. Further conversion of ketal to the corresponding ketone **8** (80% yield) by treatment with concentrated HCl, followed by a second reductive amination reaction with the protected piperazine-based compound **3**, yielded **9** (56% yield). Final deprotection of **9** gave the desired polyamine **PA3** (76% yield). The chemical identity of all compounds was confirmed by ^1^H and ^13^C NMR spectroscopy and by electrospray ionization high-resolution mass spectrometry (ESI-HRMS) (see Supporting Information (SI)). The distribution pattern of the NMR spectral data combined with X-ray diffraction analysis provide strong evidence that **PAs** adopt well-defined, rod-like structures with all the piperazine and piperidine rings in chair conformations in both solution and the solid state (Figures S5, S13, and S23).^32^

In the ^1^H NMR spectra acquired at RT in CDCl_3_, and at 400 MHz, the axial and equatorial protons of the piperidine rings (H6–6′ and H7–7′ for **PA2**; H6–6′, H7–7′, H9–9′, and H10–10′ for **PA3**) are discernible: significant chemical shift differences between axial and equatorial positions of each methylene group are evident, with axial protons shifted in the upfield region (Figures S5 and S13). Methine protons (H5 for **PA2**; H5 and H8 for **PA3**) reveal typical signal multiplicity of proton located in the axial position, featuring two axial–axial and two axial–equatorial couplings (Figures S5 and S23). Altogether, these features provide strong evidence that all piperidine rings have a single chair conformation.^32^ Moreover, the eight protons of piperazine rings (H3–3′ and H4–4′ for both **PA2** and **PA3**) appear as two broad singlets (Figures S5 and S13), suggesting a rapid exchange between the axial and equatorial arrangements on the NMR time scale through both nitrogen and chair–chair inversions. X-ray diffraction data of the protected compounds **5** and **9** also support the NMR conformational analysis of polyamines featuring a linear rod-like structure on which piperazine and piperidine rings are in chair conformations (solvent, hexane) (see SI). The most relevant feature of such compounds in the crystal lattice is the supramolecular self-assembly of each molecule stabilized by the formation of several hydrogen bonds with water molecules, as described in the SI.

**Scheme 2:**
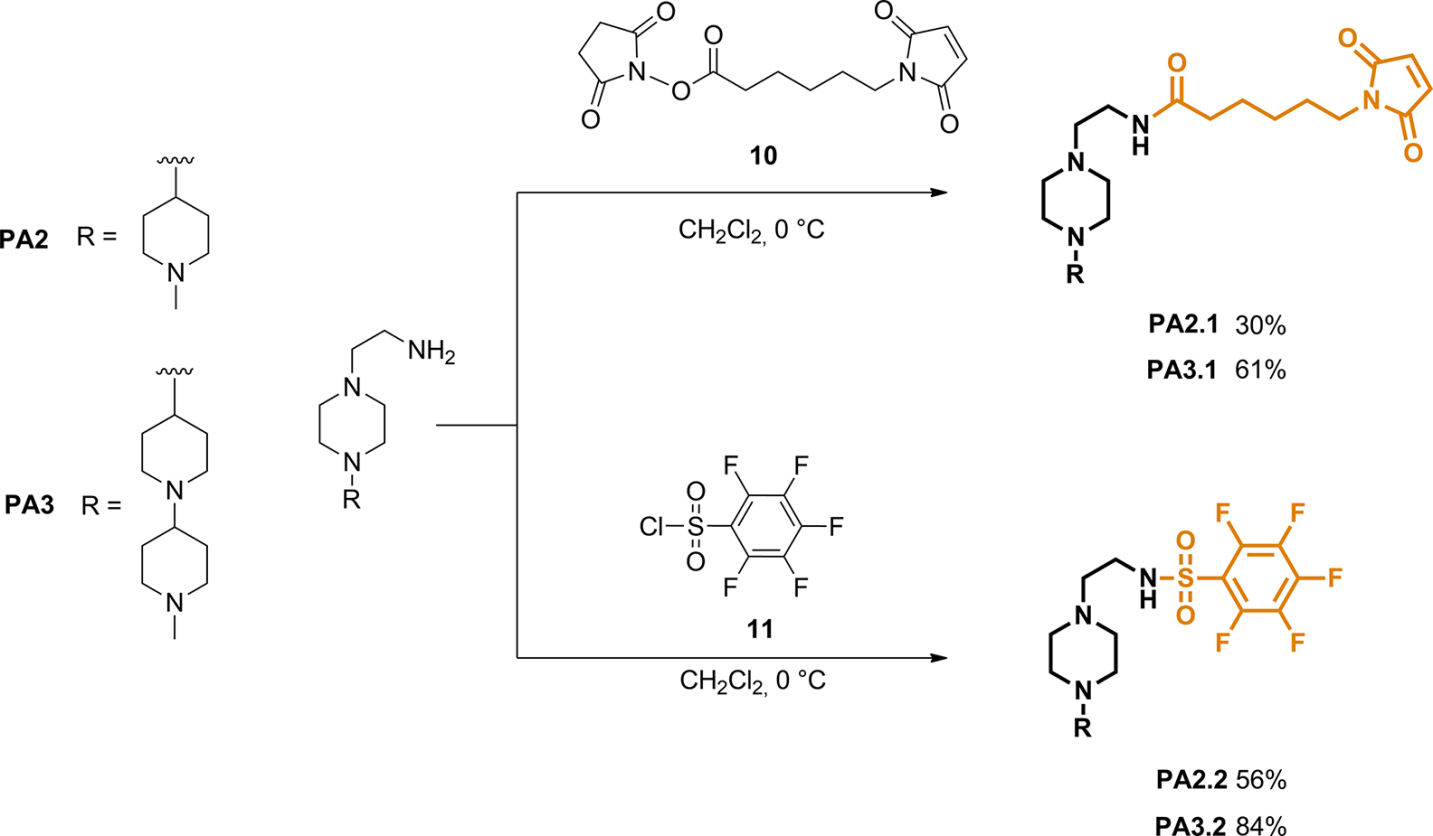
Procedures for the Synthesis of Polyamine-Thiol-Reactive Linkers (**PA2.1/2** and **PA3.1/2**)

Primary amines functionalization of **PAs** with sulfhydryl-reactive cross-linkers was also afforded in good yields. Sulfhydryl groups are useful targets for protein conjugation and labeling. Thiols are present in a large number of proteins, often linked by disulfide bonds (−S–S−) within or between polypeptide chains, but are not as numerous as primary amines; thus, cross-linking via sulfhydryl groups is more selective and precise. Maleimides remain the reagents of choice for the preparation of therapeutic and imaging protein conjugates, despite the known instability of the resulting products that undergo thiol-exchange reactions.^33−35^ In recent years, numerous other electrophiles were designed to probe cysteine residues,^33,35,36^ including haloacetyls, aziridines, acryloyls, arylating agents, vinylsulfones, pyridyl disulfides, TNB-thiols, and disulfide reducing agents. Recently, pentafluorobenzene sulfonamide end-group proved to be a versatile handle for selective functionalization of cysteine over other amino acids via nucleophilic aromatic substitution (SN_Ar_).^26,28^ Accordingly, maleimide- and fluorobenzene-based linkers were chosen to selectively conjugate polyamines to HumAfFt. By coupling the primary amines with *N*-hydroxysuccinimidyl ester-activated linker **10**, the preparation of polyamine-thiol-reactive linkers **PA2.1** and **PA3.1** was allowed in 30% and 61% yields, respectively (Scheme 2). For the synthesis of **PA2.2** and **PA3.2**, a slight excess of sulfonyl chloride reagent **11** was used affording the corresponding compounds in 56% and 84% yields, respectively (Scheme 2). A comprehensive characterization of polyamine-thiol-reactive linkers is reported in the SI.

### Design and Preparation of siRNA-PAs-HumAfFt Systems

**Scheme 3:**
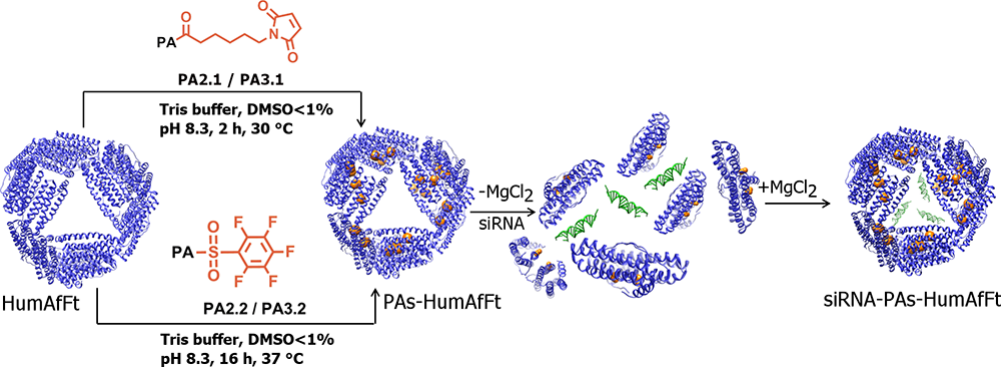
Schematic View of the Entrapment of siRNA into HumAfFt (i) On the left, chemical conjugations of HumAfFt depicted in blue cartoon to maleimide- (upper level) and pentafluorobenzene-based (lower level) compounds, both shown as orange spheres in the inner cavity of the protein; (ii) encapsulation of siRNA (depicted in green) into the **PAs-HumAfFt** favored by divalent cation-triggered assembly mechanism.

The stability and the ability of HumAfFt to reversibly assemble are crucial to incorporate negatively charged siRNA molecules, thus providing a safe vehicle for tumor cell-specific siRNA delivery. However, due to the presence of many negatively charged residues, the inner cavity of HumAfFt is not adapted to the entrapment of negative molecules. To circumvent these difficulties, a highly reactive cysteine per monomer was introduced by a point mutation as reported elsewhere.^20,37^ This mutation (M54C) in the protein inner cavity offers the opportunity to covalently conjugate up to 24 thiol-reactive linkers, such as maleimide- or pentafluorobenzenesulfonamide-based compounds bearing positively charged side-chains (**PAs**) at pH 7.4 as predicted by *ab initio* calculation (Figure S26). As illustrated in Scheme 3, chemical conjugations were performed using the assembly disassembly mechanism of HumAfFt in very mild conditions, at pH 8.3, to promote the nucleophilic reaction between thiols and **PAs** linkers. After complete removal of unreacted compounds by gel filtration chromatography, protein mass spectrometry measurements carried out on a QTof Synapt G2 confirmed the efficient functionalization of HumAfFt with one linker per monomer (Figure S27). Overall assembly was assessed by size-exclusion chromatography on the **PAs** conjugated protein in the presence of 50 mM MgCl_2_ at physiological pH. More than 80% of the HumAfFt retained its assembled 24-meric structure after the conjugation resulting stable after long storage (up to a month) at 4 °C (Figure S28). As illustrated in Scheme 3, HumAfFt was then disassembled into dimeric stable subunits by removing MgCl_2_ and mixed to a smart pool of four siRNAs in 5:1 ratio of siRNA per cage. The physical entrapment was performed by the rapid assembly upon MgCl_2_ addition. Indeed, the cation-triggered assembly favored the entrapment of siRNA, which is stabilized by the coupled effect between electrostatic interaction and physical confinement, when the 24 subunits are restored (Scheme 3). As determined by UV–vis spectroscopy, the encapsulation efficiency of **siRNA-PAs-HumAfFt** is about 50%. The oligomerization state of **siRNA-PAs-HumAfFt** was assessed by DLS measurements which clearly showed that siRNA entrapment does not affect the overall protein structure and assembly. The *Z* average values which indicate mean hydrodynamic particle diameters were (17.40 ± 0.06 nm) and (18.65 ± 0.6 nm) for **PAs-HumAfFT** and **siRNA-PAs-HumAfFt**, respectively, and are in complete agreement with human ferritin values^38^ confirming the restoring of the nanocage after the inclusion of siRNA. However, **siRNA-PAs-HumAfFt** showed an additional distribution at higher *Z* values indicating the presence of some aggregates (Figure S29).

### Stability Evaluation of siRNA-PAs-HumAfFT after RNase Digestion

**Figure 2:**
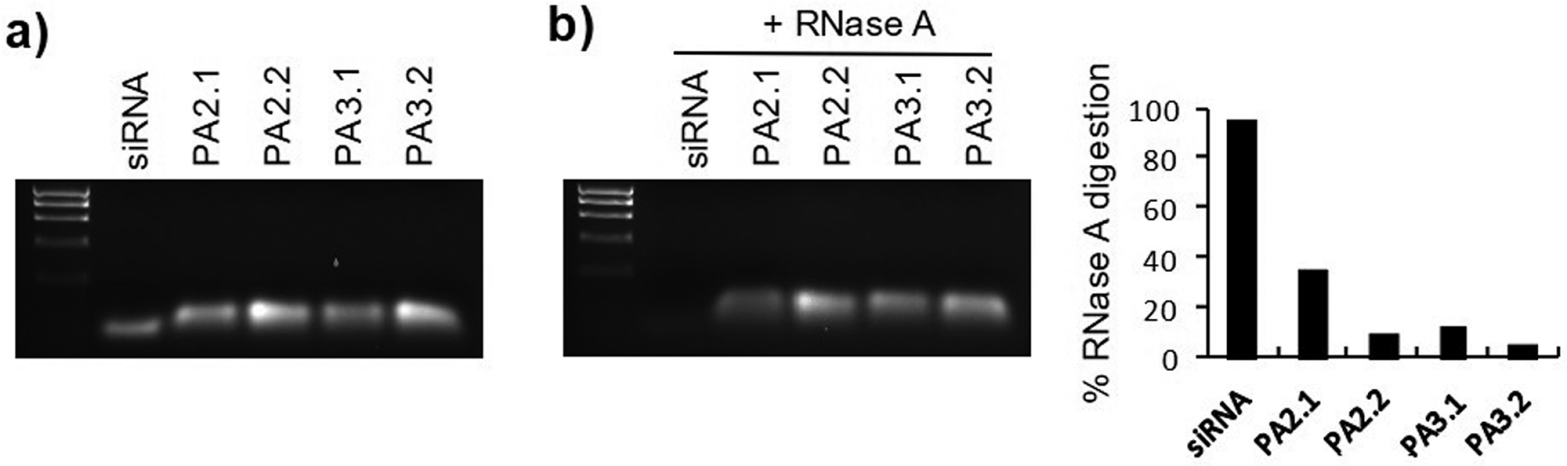
(a) Gel electrophoresis for **PAs-HumAfFt** encapsulating siRNA. Here, naked siRNA was used as a positive control in equivalent amount as **PAs-HumAfFt** complexes, prepared as described in the text. (b) RNase A digestion assay: each sample as in panel (a) was incubated with 0.5 mg/mL RNase A for 30′ at 37 °C; the left panel shows gel electrophoresis after RNase treatment; naked siRNA in equivalent amount as **PAs-HumAfFt** complexes was used as positive control; in the right panel, the histogram shows % RNase A digestion after densitometric analysis (ImageJ software) of RNase A digested samples (panel b) over undigested (panel a).

The capability of the **PAs-HumAfFt** to encapsulate and protect siRNA against nuclease degradation over time was investigated by an electrophoresis analysis on agarose gel. As shown in Figure 2a, the encapsulation of siRNA into **PAs-HumAfFt** is confirmed by a retardation shift of our samples in the gel compared to naked siRNA used as a control. Further, the efficient protection of the encapsulated siRNA was confirmed using RNase digestion assay. siRNA entrapped into conjugated ferritin nanoparticles remained intact after digestion with 0.5 mg/mL RNase A treatment for 30′ at 37 °C (Figure 2b), as compared to an equivalent amount of naked siRNA (CTRL) that, on the contrary, was 95% degraded (Figure 2b). Indeed, the **PA2.2-**, **PA3.1-**, and **PA3.2-HumAfFt** complexes reached, respectively, 91%, 87%, and 94% of siRNA protection, while approximately only 65% of siRNA in the **PA2.1-HumAfFt** complex was protected from the RNase A digestion in agreement with the predicted p*K*_a_ values of each linker.

### Cytotoxicity Evaluation of siRNA-PAs-HumAfFt

**Figure 3:**
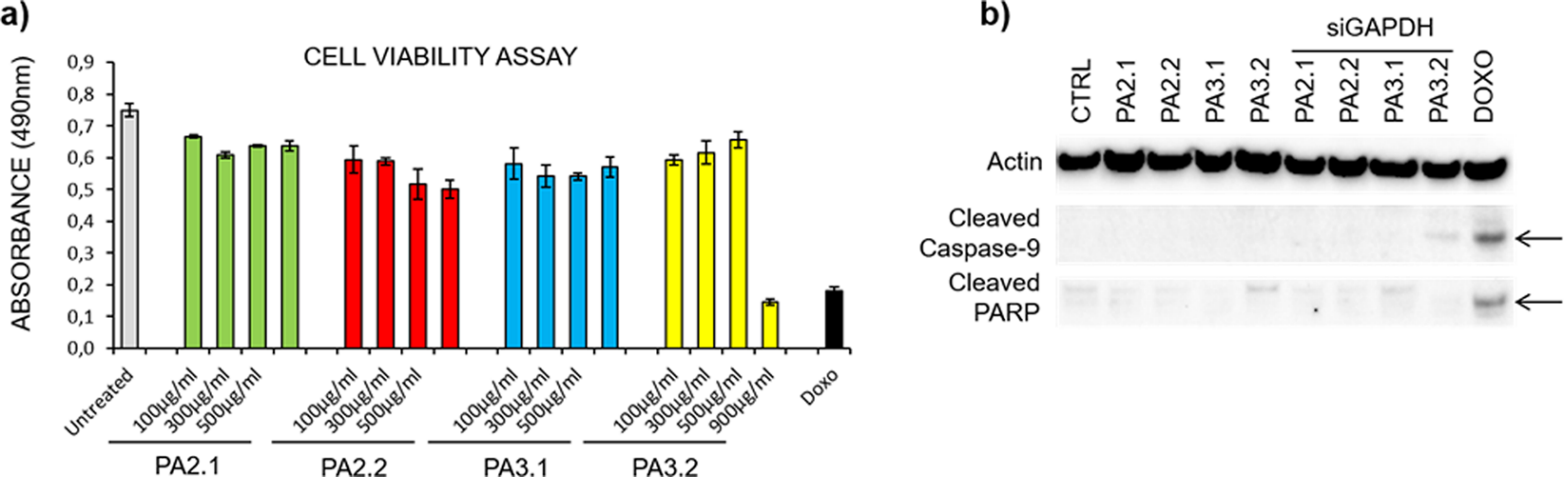
(a) Cell viability evaluation of HeLa cells left untreated or treated with the indicated constructs at the indicated concentrations for 24 h, doxorubicin 2 μM apoptotic drug was used as a positive control. Cells were processed as described in the Experimental Section for the cell viability assay. Histograms show mean from triplicates; bars indicate S.E.; *p*-value <0.05 by Student *t* test. (b) Total protein lysates were extracted from HeLa cells treated as indicated for 24 h. Cells were harvested and analyzed by immunoblotting using specific antibodies as indicated.

To evaluate the cytotoxicity effect of **siRNA-PAs-HumAfFt** systems, we performed cell viability assay on HeLa cells at four different concentrations of **PAs** conjugated to HumAfFt after 24 h, as reported in Figure 3a. Remarkably, the **PAs-HumAfFt** systems did not induce any cytotoxic effect up to 2 μM per nanoparticle (i.e., 1000 μg/mL protein) in our experimental conditions. These results displayed a negligible toxicity with respect to a treatment with 2 μM doxorubicin (1 μg/mL), an apoptotic agent used as a positive control. In addition, immunoblotting on total protein lysates was performed 24 h after treatment with **PAs-HumAfFt** conjugates, confirming that our systems did not induce programmed cell death. As clearly shown in Figure 3b, no cleaved active form of the PARP and Caspase-9 proteins, markers of cells undergoing apoptosis, can be found in **PAs-HumAfFt** treated cells as compared to the doxorubicin treatment. These results confirm that there is no cytotoxicity upon **PAs-HumAfFt** delivery.

### siRNA Delivery Assessment by Flow Cytometry Analysis

HumAfFt is recognized and internalized by the TfR1, which is overexpressed in many types of tumor cells (20). To validate the uptake efficiency and siRNA delivery to cancer cells, we performed time course experiments on HeLa cells treated with **FITC-siGAPDH-PAs-HumAfFt** and analyzed them by flow cytometry. All the experiments were carried out at 600 nM of 24-meric protein concentration for a final delivery of 300 nM of siRNA. As a control of protein uptake efficiency, cells incubated with FITC-plain-HumAfFt were used confirming a 99% of internalization under these experimental conditions. The FACS analysis is summarized in Figure S31 and show the percentage of cells internalizing **FITC-siRNA-PAs-HumAfFt**. These data confirmed that FITC-siGAPDH is delivered into HeLa cells. Interestingly, the percentages of FITC-positive cells are 24.3% and 19.6% for **PA2.2-** and **PA3.2-HumAfFt** nanoparticles, respectively. Both **PA2.1**- and **PA3.1**-**HumAfFt** display a minor efficiency (10.7% and 5.6%, respectively) compared to their analogues bearing fluorobenzenesulfonamide moiety, in line with their less performance on siRNA protection/encapsulation as shown by RNase assay in Figure 2. From the structural point of view, the increase in efficiency of fluobenzenesulfonamide linkers with respect to the maleimide ones is most probably due to the more conformationally flexible alkyl chain of the latter, which can imply an unfavorable electrostatic interaction between PAs and siRNA molecules.

### siRNA Targeted Delivery *in Vitro* and Silencing Efficacy

**Figure 4:**
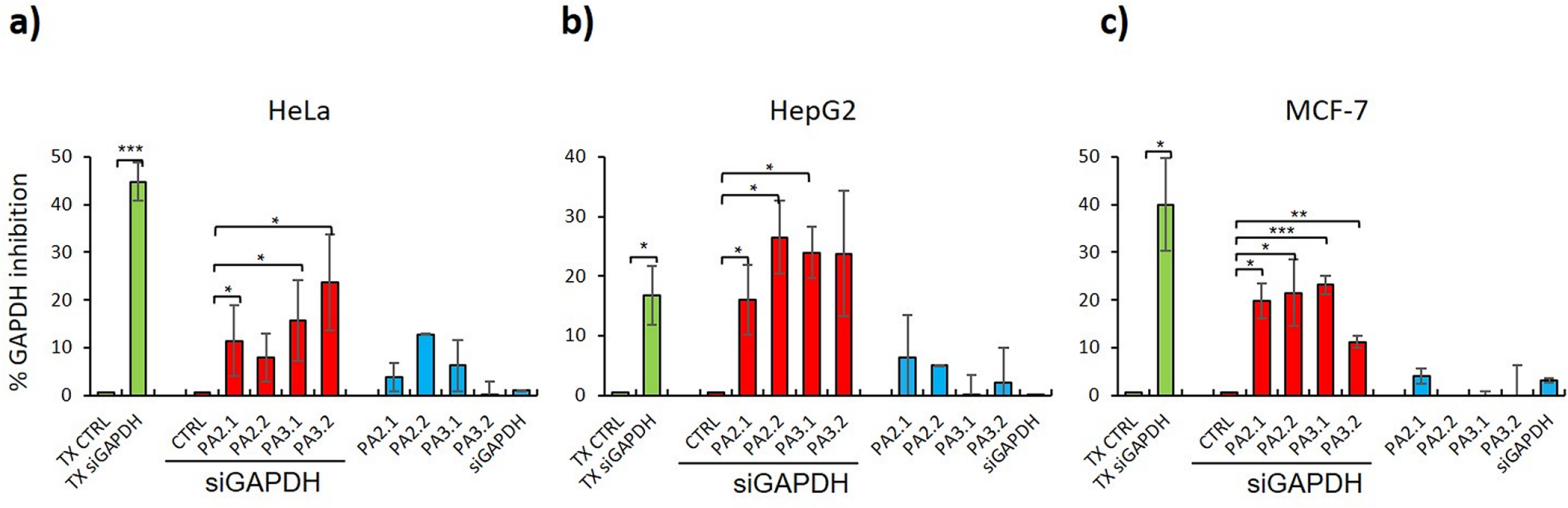
Quantification of GAPDH inhibition in HeLa (a), HepG2 (b), and MCF-7 (c) cells. Cells were transfected with LT1/TKO transfection agents (TX CTRL) and with LT1/TKO + siGAPDH (TX siGAPDH) (green columns) or treated with the indicated different **siGAPDH-PAs-HumAfFt** systems for 24 h (red columns). Untreated cells (CTRL) and cells treated with **PAs-HumAfFt** or naked siGAPDH, as indicated, were used as a control (blue columns). cDNAs were analyzed by qPCR with primers specific for GAPDH and normalized to Actin. Results are expressed as % GAPDH inhibition of transfected cells or treated cells versus relative controls. Histograms show mean from 3 independent experiments; bars indicate S.E.; asterisks indicate *P*-value (*0.01 ≤ *P<* 0.05; **0.001 ≤ *P<* 0.01; ***P< 0.001).

Once we had assessed that our systems confer siRNA protection, are not cytotoxic, and are able to enter HeLa cells and deliver FITC-siGAPDH, the ability of the **PAs-HumAfFt** to deliver its nucleic acid cargo and efficacy of silencing was investigated in three cell lines, namely, HeLa, HepG2, and MCF-7, which overexpress CD71 receptor as demonstrated by immunoblotting analysis described in section 7 of SI (Figure S30). To evaluate intracellular release and gene knockdown efficiency, cells were treated over 24 h with siRNA against GAPDH encapsulated into **PAs-HumAfFt** conjugates, and the silencing effect was compared with traditional transfection method using the commercial agent LT1/TKO (Figure 4, green columns) as a reference. The GAPDH gene is a key regulatory enzyme of glycolysis that was chosen for its stability and constitutive expression at high levels in most tissues and cells, and for its constant expression in the cells under investigation; these characteristics allowed us to evaluate siRNA entry and consequent inhibition of GAPDH production, ensuring that the observed downregulation of GAPDH expression levels is only due to siRNA delivery into cells by **PAs-HumAfFt**. As expected, naked siGAPDH exhibited a negligible amount of uptake due to its inability to cross the cell membrane (Figure 4a−c blue columns and Figure S31). Similarly, the **PAs-HumAfFt** formulations used as negative control did not induce any substantial silencing effect in all the tested cell lines (Figure 4a–c, blue columns).

Remarkably, the **siRNA-PAs-HumAfFt** systems provided an excellent cellular delivery able to effectively induce specific GAPDH silencing in targeted cells, demonstrating siRNA protection and release into the cytoplasm. In HeLa cells, our system could achieve 24% of silencing with **PA3.2**, 19% with **PA3.1**, and 16% with **PA2.1** (Figure 4a, red columns), while **PA2.2-HumAfFt** was less efficient but still significant in comparison to other systems^15,27^ and traditional transfection agents (Figure 4a, green columns). In HepG2 cells (Figure 4b), which usually achieve a very low rate of transfection efficiency,^39^ our carriers could achieve a 26.5% knockdown of GAPDH with **PA2.2-HumAfFt**, 24% with **PA3.1-HumAfFt**, and 23.7% with **PA3.2-HumAfFt** (Figure 4b, red columns). In HepG2 cells, these carriers exhibited a better silencing efficiency as compared to the 16.7% inhibition obtained with the siRNA delivery by traditional transfection systems (Figure 4b, green columns), with the only exception of **PA2.1-HumAfFt** (16%), which show a similar knockdown efficiency with respect to traditional systems. The GAPDH silencing of **PAs-HumAfFt** in HepG2 transfected cells appeared to be slightly proportional to the number of protonated amines. Based on computational predictions, **PA2.1** exhibited only one positive charge at pH 7.4 matching on the piperazine ring making siRNA entrapment into HumAfFt not optimal even though the nucleic acid to protein ratio was kept constant among the four delivery systems. As shown in Figure 4c (red columns), **siGAPDH-PAs-HumAfFt** formulations exhibited a satisfactory knockdown of GAPDH also in MCF-7 cells compared to LT1/TKO transfection agents. **PA2.1-**, **PA2.2-**, and **PA3.1-HumAfFt** exhibited a silencing effect higher than 20% (20%, 21.4%, and 23%, respectively), with the only exception of **PA3.2-HumAfFt**. As an interesting observation of this work, the pentafluorobenzene-based one emerged as an extremely valid alternative thiol-reactive group to the most widely used maleimide-based group displaying an easy and complete conjugation of both **PA2.2** and **PA3.2** compounds to cysteine residues and a good ability to deliver siGAPDH into three cell lines. Regarding the **PA3.2** compound, the combination of pentafluorobenzene moiety and an additional piperidine ring gives rise to a totally soluble linker in aqueous solutions compared to its maleimide-based analogues usually soluble only in polar organic solvents. As such, **PA3.2** displayed a notable effect in HeLa and in HepG2 cells compared to the others. The observed differences of delivery into cells among the different **siGAPDH-PAs-HumAfFt** are at least in part ascribable to the variability of cell cultures *in vitro*. Indeed, variable expression levels of TfR1 among HeLa, MCF-7, and HepG2 cells could influence the **siGAPDH-PAs-HumAfFt** entry efficiency into cells and the consequent rate of GAPDH inhibition (Figure S30). According to previously reported studies,^20^ the mechanism by which **PAs-HumAfFt** systems enter the cells may be based on the clathrin-mediated endocytosis, the major route for the internalization of HumAfFt through the TfR1 receptor.^16,40^ Upon intracellular internalization, the complex is expected to be contained into early endosomes where the TfR1 is released to be recycled back to the surface. The ability of HumAfFt to bind TfR1 is crucial for siRNA uptake in cell lines resistant to transfection with traditional methodologies taking advantage that cancer cells are naturally avid of iron in response to higher proliferating rates. As a result, we demonstrated that siRNA-**PAs-HumAfFt** nanoparticles can deliver and protect siGAPDH from degradation through TfR1 receptor and exhibited a significant silencing of GAPDH gene expression *in vitro* in three different cancer cell lines. The observed minor effect of **siGAPDH-PAs-HumAfFt** in HeLa cells can be addressed to a significant lower TfR1 expression, i.e., 2.5 times less than HepG2 and MCF-7 cells. However, the intracellular release of siGAPDH from our systems was confirmed by qPCR measurements, which overall indicate more than 25% of GAPDH silencing. Further studies will be necessary to probe the exact mechanism forming the basis of how the endosomal escape is achieved and to what extent it can affect the release of the cargo.^16,41,42^ Nevertheless, our data clearly indicate that unmodified siRNA can be successfully delivered through **PAs-HumAfFt** conjugates and released into the cytosol after internalization via the TfR1 receptor.

## Conclusions

In summary, we developed a targetable siRNA delivery system based on engineered HumAfFt efficiently functionalized with novel piperazine-based compounds featuring one or two piperidine rings. These rigid-rod-like amines linked to thiol-reactive reagents (i.e., maleimide and fluorobenzenesulfonamide) were rationally designed and synthesized for chemoselective conjugation of a topologically selected cysteine residue located inside the HumAfFt cavity. Notably, pentafluorobenzene-based derivatives bearing an electron-withdrawing para substituent ensured thiol-selective modification of HumAfFt similarly to maleimide-based reactive groups. The unique divalent-cation-triggered oligomerization properties of HumAfFt were unaltered after the conjugation and were exploited to host and stabilize negatively charged siRNA molecules. Despite the constant development of increasingly efficient gene carriers based on protein nanoparticles functionalized with various organic polymers,^27,43^ the proposed siRNA delivery system represents a useful and straightforward platform to encapsulate various RNA therapeutic agents with satisfactory levels of silencing representing a valid alternative in the case of cells particularly difficult to transfect, such as HepG2.^44^ By using a receptor-driven uptake, the ferritin-based nanocarriers are naturally targeted systems, stable in the extracellular environment, thus providing a unique nanotechnological tool designed to protect siRNA from degradation and to prevent the formation of aggregates unsuitable to the rigorous demands of therapeutic manufacturing.^45^ As future work, **PAs-HumAfFt** conjugates may be further modified to use multiple trafficking pathways and to improve the release of siRNA from endosomal compartments.^46−48^

## Experimental Section

### Materials and Methods

Melting points were taken in open capillaries on a Büchi Melting Point B-545 apparatus and are presented uncorrected. ^1^H NMR and ^13^C NMR spectra were recorded using a Bruker 400 Ultra ShieldTM spectrometer (operating at 400 MHz for ^1^H and 100 MHz for ^13^C). High-resolution mass spectra (HRMS) were recorded on Bruker BioApex Fourier transform ion cyclotron resonance (FT-ICR) mass spectrometer. Chemical shifts are reported in parts per million (ppm). Multiplicities are reported as follows: singlet (s), doublet (d), triplet (t), multiplet (m), triplet of doublets (td), triplet of triplets (tt), and quartet of doublets (qd).

### Production and Purification of HumAfFt

HumAfFt was designed with a M54C mutation per monomer to functionalize the protein inner cavity with sulfhydryl-reactive polyamines, expressed and purified as previously described.^20^ Briefly, cells were grown at 37 °C to OD600 of 0.6 in LB medium containing 100 μg/mL of kanamycin, and protein expression was induced by 1 mM IPTG for 3 h at 37 °C. Cells harvested by centrifugation were resuspended in 20 mM HEPES buffer, pH 7.5, containing 300 mM NaCl, 1 mM TCEP, and a Complete Mini Protease Inhibitor Cocktail Tablet (Roche). Cells were disrupted by sonication and the soluble fraction was purified by heat treatment. Denatured *E. coli* proteins were removed by centrifugation at 15 000 rpm at 4 °C for 1 h. The soluble protein was further purified by ammonium sulfate precipitations. The precipitated fraction was resuspended and dialyzed in 20 mM HEPES, 50 mM MgCl_2_, and pH 7.5 (Buffer A). As a final purification step, the protein was loaded onto a HiLoad 26/600 S400 column by using an ÄKTA-Pure system (GE Healthcare). Purified protein concentration in the 24-meric conformation was calculated by measuring the UV spectrum using an extinction coefficient of 777 400 M^–1^cm^–1^.

### Synthesis of Polyamine-Thiol-Reactive Linkers

#### Synthesis of 2-(4-(1-Methylpiperidin-4-yl)piperazin-1-yl)ethanamine (**PA2**)

Polyamine **PA2** was synthesized via reductive amination reaction using NaBH(OAc)_3_ (sodium triacetoxyborohydride) as a mild reducing agent (Scheme 1a). *First step*: ethyl trifluoroacetate (**2**) (15.46 mmol, 2.19 g) was added to a solution of 2-(piperazin-1-yl)ethanamine (**1**) (7.73 mmol, 1 g) in acetonitrile (154.6 mL) at 0 °C. The reaction was stirred for 4 h at RT. Afterwards, the solution was evaporated under reduced pressure and 2,2,2-trifluoro-*N*-(2-(piperazin-1-yl)ethyl)acetamide (**3**) was obtained in quantitative yield (2.25 g, 10 mmol). *Second step*: to a stirred solution of **3** (3.1 mmol, 695.4 mg) and 1-methylpiperidin-4-one (**4**) (4.41 mmol, 500 mg) in dry 1,2-dichloroethane (13.16 mL) at RT, NaBH(OAc)_3_ (6.186 mmol, 1.31 g) and AcOH (0.253 mL) were added. The mixture was stirred for 12 h, and the resulting suspension was filtered under vacuum. After the solvent evaporation, the crude material was purified by column chromatography on Al_2_O_3_, using chloroform as eluent. Compound **5** was obtained in 60% yield (2.64 mmol, 852 mg) as a white solid. *Third step*: a mixture of **5** (0.344 mmol, 111 mg) and 5% aq. K_2_CO_3_ (1.4 mL) in methanol (3.4 mL) was refluxed for 24 h. After removal of methanol under reduced pressure, the mixture was extracted with chloroform. The organic layer was washed with brine, dried over Na_2_SO_4_, and evaporated under reduced pressure. Compound **PA2** was obtained in 75% yield (0.258 mmol, 58 mg) as a yellow oil.

#### Synthesis of 2-(4-(1′-Methyl-[1,4′-bipiperidin]-4-yl)piperazin-1-yl)ethanamine (**PA3**)

Similarly, the synthesis of polyamine **PA3** was performed by iterative reductive amination reaction (Scheme 1b). *First step*: to a stirred solution of **4** (8.83 mmol, 1 g) and 1,4-dioxa-8-azaspiro[4.5]decane (**5**) (8.83 mmol, 1.262 g) in 1,2-dichloroethane dry (10.32 mL), NaBH(OAc)_3_ (12.35 mmol, 2.6 g), and AcOH (0.253 mL) were added at RT. The mixture was stirred for 12 h, and the resulting suspension was filtered under vacuum. After the evaporation of the solvent, the crude material was purified by column chromatography on Al_2_O_3_, using chloroform as eluent. Compound **7** was obtained in 29% yield (2.56 mmol, 614 mg) as a yellow oil. *Second step*: compound **7** (2 mmol, 480 mg) was treated with concentrated hydrochloric acid (21 mL) at 0 °C and then allowed to warm to RT. After 2 h, 77 mL of dichloromethane were added to the mixture at 0 °C, followed by aqueous NaOH solution to reach pH = 14. The organic phase was dried over Na_2_SO_4_, and the solvent was evaporated. The crude material was purified by column chromatography on Al_2_O_3_, using chloroform as eluent. Compound **8** was obtained in 80% yield (1.6 mmol, 313.6 mg) as a yellow oil. *Third step*: to a stirred solution of **8** (3.09 mmol, 607 mg) and **3** (2.16 mmol, 484 mg) in dry 1,2-dichloroethane (9.2 mL), NaBH(OAc)_3_ (3.67 mmol, 778 mg), and AcOH (0.150 mL) were added at RT. The mixture was stirred for 12 h, and the resulting suspension was filtered under vacuum. After the evaporation of solvent, the crude material was purified by column chromatography on Al_2_O_3_, using chloroform as eluent. Compound **9** was obtained in 56% yield (1.73 mmol, 71.67 mg) as a white solid. *Fourth step*: a mixture of **9** (0.572 mmol, 232 mg) and 5% aq. K_2_CO_3_ (2.28 mL) in methanol (5.72 mL) was refluxed for 24 h. After removal of methanol under reduced pressure, the mixture was extracted with chloroform. The organic layer was washed with brine, dried over Na_2_SO_4_, and evaporated under reduced pressure. Compound **PA3** was obtained in 76% yield (0.434 mmol, 134 mg) as a yellow oil.

#### Synthesis of 6-(2,5-Dioxo-2,5-dihydro-1*H*-pyrrol-1-yl)-*N*-(2-(4-(1-methylpiperidin-4-yl)piperazin-1-yl)ethyl)hexanamide (**PA2.1**) and 6-(2,5-Dioxo-2,5-dihydro-1*H*-pyrrol-1-yl)-*N*-(2-(4-(1′-methyl-[1,4′-bipiperidin]-4-yl)piperazin-1-yl)ethyl)hexanamide (**PA3.1**)

Preparation of maleimide-based compounds was performed by reacting *N-*hydroxysuccinimide ester-activated cross-linker with the primary amine of polyamines (Scheme 2). A solution of **PA2** (0.273 mmol, 62 mg) in CH_2_Cl_2_ (0.407 mL) was cooled at 0 °C, and 2,5-dioxopyrrolidin-1-yl-6-(2,5-dioxo-2,5-dihydro-1*H*-pyrrol-1-yl)hexanoate (**10**) was added in slight excess. After 15 min, the resulting solution was evaporated under reduced pressure. Precipitation in hexane afforded compound **PA2.1** in 30% yield (0.0819 mmol, 34.34 mg) as a yellow solid.

Similarly, a solution of **PA3** (0.162 mmol, 50 mg) in CH_2_Cl_2_ (0.241 mL) was cooled at 0 °C, and linker **10** was added in a molar ratio of 1:1 (0.162 mmol, 49.94 mg). After 30 min, the resulting solution was evaporated under reduced pressure. The crude material was purified by column chromatography on Al_2_O_3_, using chloroform as eluent, and compound **PA3.1** was obtained in 61% yield (0.098 mmol, 49 mg) as a white solid.

#### Synthesis of 2,3,4,5,6-Pentafluoro-*N*-(2-(4-(1-methylpiperidin-4-yl)piperazin-1-yl)ethyl)benzenesulfonamide (**PA2.2**) and 2,3,4,5,6-Pentafluoro-*N*-(2-(4-(1′-methyl-[1,4′-bipiperidin]-4-yl)piperazin-1-yl)ethyl)benzenesulfonamide (**PA3.2**)

Preparation of fluorobenzene-based compounds was performed by reacting the sulfonyl chloride reagent with the primary amine of polyamines (Scheme 2). Compound **PA2** (0.309 mmol, 70 mg) was added to a solution containing a slight excess of 2,3,4,5,6-pentafluorobenzene-1-sulfonyl chloride (**11**) (0.463 mmol, 68 μL, *d* = 1.796 g/mL) in CH_2_Cl_2_ (4.63 mL). The reaction was stirred at 0 °C for 30 min. The solvent was evaporated under reduced pressure, and the crude material was purified by precipitation in hexane. Compound **PA2.2** was obtained in 56% yield (0.173 mmol, 78.98 mg) as a white solid. Similarly, **PA3** (0.129 mmol, 40 mg) was added to a solution of **11** (0.193 mmol, 628 μL) in CH_2_Cl_2_ (1.29 mL). The reaction was stirred at 0 °C for 30 min. The solvent was evaporated under reduced pressure, and the crude residue was purified by precipitation in hexane. Compound **PA3.2** was obtained in 84% yield (0.108 mmol, 58.3 mg) as a white solid.

### Chemical Characterization of Compounds

#### 2,2,2-Trifluoro-*N*-(2-(piperazin-1-yl)ethyl)acetamide (**3**)

Yellow solid; mp 88 °C. ^1^H NMR (400 MHz, CDCl_3_, 298 K): δ 7.12 (broad s, 1H, −CONH−), 3.41 (pseudo t, 2H, H2), 2.88 (t, *J* = 4.9 Hz, 4H, H4–4′), 2.53 (t, *J* = 6 Hz, 2H, H3), 2.44 (broad s, 4H, H5–5′), 1.92 (s, 1H, −NH−). ^13^C NMR (101 MHz, CDCl_3_, 298 K): δ 157.2 (q, *J* = 37.16 Hz), 117.3, 114.4, 56.0, 54.0, 46.1, 36.0. ESI-HRMS *m*/*z* calcd for C_8_H_15_F_3_N_3_O: 226.11617, found 226.116640 [M + H]^+^.

#### 2,2,2-Trifluoro-*N*-(2-(4-(1-methylpiperidin-4-yl)piperazin-1-yl)ethyl)acetamide (**5**)

White solid (yield 60%); mp 86 °C. ^1^H NMR (400 MHz, CDCl_3_, 298 K): δ 7.11 (broad s, 1H, −CONH−), 3.39 (pseudo t, 2H, H1), 2.89 (broad d, *J* = 11.6 Hz, 2H, H7eq–7′eq), 2.56–2.49 (m, 10H, H2, H3–3′, H4–4′), 2.24 (s, 3H, H8), 2.21 (tt, *J* = 11.4 Hz, *J* = 3.7 Hz, 1H, H5), 1.93 (td, *J* = 11.9 Hz, *J* = 1.8 Hz, 2H, H7a–7′a), 1.78 (broad d, *J* = 12.4 Hz, 2H, H6eq–6′eq), 1.56 (qd, *J* = 12.2 Hz, *J* = 3.6, 2H, H6a–6′a). ^13^C NMR (101 MHz, CDCl_3_,298 K): δ 157.1 (q, *J* = 36.8 Hz), 116.0 (q, *J* = 287.7 Hz), 61.4, 55.4, 55.4, 53.2, 49.0, 46.1, 36.2, 28.2. ESI-HRMS *m*/*z* calcd for C_14_H_26_F_3_N_4_O: 323.20532, found 323.20528 [M + H]^+^.

#### 2-(4-(1-Methylpiperidin-4-yl)piperazin-1-yl)ethanamine (**PA2**)

Yellow oil (yield 75%). ^1^H NMR (400 MHz, CDCl_3_, 298 K): δ 2.87 (broad d, *J* = 11.7 Hz, 2H, H7eq–7′eq), 2.76 (t, *J* = 6.2 Hz, 2H, H1), 2.57 (br s, 4H, H4–4′), 2.47 (broad s, 4H, H3–3′), 2.40 (t, *J* = 6.2 Hz, 2H, H2), 2.23 (s, 3H, H8), 2.19 (tt, *J* = 11.5 Hz, *J* = 3.7 Hz, 1H, H5), 1.93–1.88 (m, 4H, H7a–7′a, −NH_2_), 1.78 (broad d, *J* = 12.5 Hz, 2H, H6eq–6′eq), 1.56 (qd, *J* = 12.2 Hz, *J* = 3.5 Hz, 2H, H6a–6′a). ^13^C NMR (101 MHz, CDCl_3_, 298 K): δ 61.5, 60.9, 55.5, 53.6, 49.1, 46.2, 38.8, 28.2. ESI-HRMS *m*/*z* calcd per C_12_H_27_N_4_: 227.22302, found 227.22282 [M + H]^+^.

#### 8-(1-Methylpiperidin-4-yl)-1,4-dioxa-8-azaspiro[4.5]decane (**7**)

Yellow oil (yield 29%). ^1^H NMR (400 MHz, CD_3_OD, 298 K): δ 3.93 (s, 4H, H1–1′), 2.93 (broad d, *J* = 12.1 Hz, 2H, H6eq–6′eq), 2.67 (pseudo t, *J* = 5.2 Hz, 4H, H3–3′), 2.34 (tt, *J* = 11.7 Hz, *J* = 3.7 Hz,1H, H4), 2.25 (s, 3H, H7), 2.02 (td, *J* = 12.2 Hz, *J* = 2.3 Hz, 2H, H6a–6′a), 1.88–1.84 (m, 2H, H5eq–5′eq), 1.72 (pseudo t, *J* = 5.7 Hz, 4H, H2–2′), 1.58 (qd, *J* = 12.5 Hz, *J* = 3.8 Hz, 2H, H5a–5′a). ^13^C NMR (101 MHz, CD_3_OD, 298 K): δ 107.9, 65.2, 62.2, 56.2, 48.0, 46.0, 35.6, 28.6. ESI-HRMS *m*/*z* calcd for C_13_H_24_N_2_O_2_: 241.19105, found 241.19116 [M + H]^+^.

#### 1′-Methyl-[1,4′-bipiperidin]-4-one (**8**)

Yellow oil. ^1^H NMR (400 MHz, CDCl_3_, 298 K): δ 2.92 (broad d, *J* = 11.9 Hz, 2H, H5eq–5′eq), 2.82 (t, *J* = 6 Hz, 4H, H2–2′), 2.48–2.37 (m, 5H, H1–1′, H3), 2.27 (s, 3H, H6), 1.98 (td, *J* = 11.6 Hz, *J* = 2 Hz, 2H, H5a–5′a), 1.79–1.75 (m, 2H, H4eq–4′eq), 1.64 (qd, *J* = 12 Hz, *J* = 3.6 Hz, 2H, H4a–4′a). ^13^C NMR (101 MHz, CDCl_3_, 298 K): δ 209.4, 60.7, 55.3, 49.0, 45.9, 41.9, 28.3. ESI-HRMS *m*/*z* calcd for C_11_H_20_NO_2_: 197.16484, found 197.16476 [M + H]^+^.

#### 2,2,2-Trifluoro-*N*-(2-(4-(1′-methyl-[1,4′-bipiperidin]-4-yl)piperazin-1-yl)ethyl)acetamide (**9**)

White solid (yield 56%); mp 102 °C. ^1^H NMR (400 MHz, CDCl_3_, 298 K): δ 7.09 (broad s, 1H, −CONH−), 3.48–3.33 (m, 2H, H1), 2.95 (broad d, *J* = 11.1 Hz, 2H, H7eq–7′eq), 2.89 (broad d, *J* = 11.2 Hz, 2H, H10eq–10′eq), 2.56–2.48 (m, 10H, H2, H3–3′, H4–4′), 2.30–2.15 (m, 7H, H5, H8, H7a–7′a, H11), 1.92 (t, *J* = 11.6 Hz, 2H, H10a–H10′a), 1.77 (pseudo t, *J* = 12.8 Hz, 4H, H6eq–6′eq, H9eq–9′eq), 1.63–1.49 (m, 4H, H6a–H6′a, H9a–9′a). ^13^C NMR (101 MHz, CDCl_3_, 298 K): δ 157.1 (q, *J* = 36.3 Hz), 115.9 (q, *J* = 288.8 Hz), 62.2, 61.6, 55.6, 55.4, 53.2, 48.9, 48.8, 46.2, 36.1, 28.4, 27.9. ESI-HRMS *m*/*z* calcd for C_19_H_35_F_3_N_5_O: 406.27882, found 406.27902 [M + H]^+^.

#### 2-(4-(1′-Methyl-[1,4′-bipiperidin]-4-yl)piperazin-1-yl)ethanamine (**PA3**)

Yellow oil. ^1^H NMR (400 MHz, CDCl_3_, 298 K): δ 2.95 (broad d, *J* = 11.6 Hz, 2H, H7eq–7′eq), 2.88 (broad d, *J* = 11.7 Hz, 2H, H10eq–10′eq), 2.78 (t, *J* = 6.4 Hz, 2H, H1), 2.57 (broad s, 4H, H3–3′), 2.47 (broad s, 4H, H4–4′), 2.40 (t, *J* = 6.4 Hz, 2H, H2), 2.30–2.14 (m, 7H, H5, H8, H7a–7′a, H11), 1.91 (pseudo t, *J* = 11.6 Hz, 2H, H10a–10′a), 1.77 (pseudo t, *J* = 13.6 Hz, 4H, H6eq–6′eq, H9eq–9′eq), 1.63–1.48 (m, 4H, H6a–6′a, H9a–9′a). ^13^C NMR (101 MHz, CDCl_3_, 298 K): δ 62.3, 61.6, 61.0, 55.6, 53.7, 49.0, 48.9, 46.2, 38.8, 28.4, 28.0. HRMS *m*/*z* calcd for C_17_H_36_N_5_: 310.29652, found 310.29657 [M + H]^+^.

#### 6-(2,5-Dioxo-2,5-dihydro-1*H*-pyrrol-1-yl)-*N*-(2-(4-(1-methylpiperidin-4-yl)piperazin-1-yl)ethyl)hexanamide (**PA2.1**)

Yellow solid (yield 30%); mp 92 °C. ^1^H NMR (400 MHz, CDCl_3_, 298 K): δ 6.68 (s, 2H, H1–2), 6.08 (s, 1H, −CONH−), 3.50 (t, *J* = 7.2 Hz, 2H, H3), 3.34–3.33 (m, 2H, H9), 3.07 (broad d, *J* = 11.2 Hz, 2H, H15eq–15′eq), 2.69–2.48 (m, 10H, H10, H11–11′, H12–H12′), 2.41–2.32 (m, 4H, H13, H16), 2.16 (t, *J* = 7.6 Hz, 2H, H7), 1.88 (broad d, *J* = 11.6 Hz, 2H, H15a–15′a), 1.79–1.71 (m, 2H, H14eq–14′eq), 1.68–1.55 (m, 4H, H4, H6), 1.34–1.24 (m, 4H, H14a–H14′a, H5). ^13^C NMR (101 MHz, CDCl_3_, 298 K): δ 171.8, 169.9, 133.2, 78.6, 55.7, 52.1, 47.9, 44.3, 36.8, 35.5, 34.8, 28.8, 27.4, 26.2, 25.5, 24.2. ESI-HRMS *m*/*z* calcd for C_22_H_38_N_5_O_3_: 420.29692, found 420.29714 [M + H]^+^.

#### 2,3,4,5,6-Pentafluoro-*N*-(2-(4-(1-methylpiperidin-4-yl)piperazin-1-yl)ethyl)benzenesulfonamide (**PA2.2**)

White solid; mp 78 °C. ^1^H NMR (400 MHz, (CD_3_)_2_SO, 298 K): δ 3.12 (t, *J* = 6.2 Hz, 2H, H1), 2.77 (broad d, *J* = 11.4 Hz, 2H, H7eq–7′eq), 2.35–2.17 (m, 10H, H2, H3–3′, H4–4′), 2.13 (s, 3H, H8), 2.02 (tt, *J* = 11.2 Hz, *J* = 3.4 Hz, 1H, H5), 1.83 (t, *J* = 11.1 Hz, 2H, H7a–H7′a), 1.62 (broad d, *J* = 11.9 Hz, 2H, H6eq–6′eq), 1.34 (qd, *J* = 11.8 Hz, *J* = 3.3 Hz, 2H, H6a–6′a). ^13^C NMR (101 MHz, (CD_3_)_2_SO, 298 K): δ 145.7, 142.8, 138.9, 136.5, 60.5, 57.0, 54.7, 53.0, 48.3, 45.7, 27.5. ESI-HRMS *m*/*z* calcd for C_18_H_26_F_5_N_4_O_2_S: 457.16911, found 457.16888 [M + H]^+^.

#### 6-(2,5-Dioxo-2,5-dihydro-1*H*-pyrrol-1-yl)-*N*-(2-(4-(1′-methyl-[1,4′-bipiperidin]-4-yl)piperazin-yl)ethyl)hexanamide (**PA3.1**)

White solid; mp 98 °C. ^1^H NMR (400 MHz, CDCl_3_, 298 K): δ 6.67 (s, 2H, H1, H2), 6.04 (broad s, 1H, −CONH−), 3.49 (t, *J* = 6.8 Hz, 2H, H3), 3.37–3.31 (m, 2H, H9), 3.03 (pseudo t, *J* = 12 Hz, 4H, H15eq–15′eq, H18eq–18′eq), 2.65–2.42 (m, 10H, H10, H11–11′, H12–12′), 2.41–2.24 (m, 7H, H13, H15a–15′a, H16, H19), 2.22–2.04 (m, 4H, H7, H18a–18′a), 1.99–1.53 (m, 12H, H4, H6, H14eq–14′eq, H14a–14′a, H17eq–17′eq, H17a–17′a), 1.39–1.22 (m, 2H, H5). ^13^C NMR (101 MHz, CDCl_3_): δ 172.8, 170.9, 134.2, 56.7, 54.8, 53.1, 48.9, 45.5, 37.7, 36.5, 35.8, 29.8, 28.4, 27.6, 27.0, 26.5, 25.2. ESI-HRMS *m*/*z* calcd for C_27_H_47_N_6_O_3_: 503.37042, found 503.37018 [M + H]^+^.

#### 2,3,4,5,6-Pentafluoro-*N*-(2-(4-(1′-methyl-[1,4′-bipiperidin]-4-yl)piperazin-1-yl)ethyl)benzenesulfonamide (**PA3.2**)

White solid (yield 84%); mp 99 °C. ^13^C NMR (101 MHz, D_2_O): δ 145.1, 142.6, 138.7, 136.2, 113.6, 65.2, 58.7, 58.0, 55.0, 52.1, 50.5, 47.9, 46.1, 42.4, 37.2, 24.6, 23.9, 13.6. ESI-HRMS *m*/*z* calcd for C_23_H_35_F_5_N_5_O_2_S: 540.24261, found 540.24312 [M + H]^+^.

### Preparation of PAs-HumAfFt Conjugates

A solution of 12 μM HumAfFt (6 mg mL^–1^ in 20 mM HEPES pH 7.5) was reduced by 2.9 mM TCEP (Tris(2-carboxyethyl)phosphine hydrochloride)) (i.e., 10 excess per SH group) for 1 h at room temperature under mild agitation. The reducing agent was then removed by gel filtration chromatography carried out according to the manufacturer’s instructions in the same buffer solution (PD10 Desalting columns, GE Healthcare). The eluted protein concentration was calculated by measuring UV_280_ absorbance and using an ε = 777 400 M^–1^ cm^–1^. Polyamines were solubilized in DMSO except for **PA3.2**, which is soluble in water, at a final concentration of 50 mM and added to 1 mL protein solution under mild agitation using a 10:1 ratio with respect to the SH group. Under these reaction conditions, the final DMSO content was <5%, which guarantees the natural folding of HumAfFt. The maleimide-based compounds (i.e., **PA2.1** and **PA3.1**) reacted with HumAfFt for 2 h at 30 °C. Conversely, **PA2.2** and **PA3.2** compounds were reacted with HumAfFt for 16 h at 37 °C as reported by Embaby et al.^26^ The samples were centrifuged 10 min at 14 000 rpm and passed through PD10 desalting columns to remove unreacted linker and residual DMSO. Protein concentration was calculated by UV–vis measurements as previously reported. The conjugation reaction was assessed by QT of Synapt G2-Si mass spectrometry analysis as reported in the SI (Figure S27). To assess the oligomerization state of the **PA**-linked HumAfFt samples, they were loaded on a gel filtration column after the addition of 50 mM MgCl_2_ on a HiLoad 16/600 Superdex 75 pg equilibrated in buffer A to confirm the molecular weight of the 24-meric structure (Figure S28) (AKTA Pure 25, GE-Healthcare). The calibration curve was calculated by measuring the retention time of conventional standards including human ferritin (MW = 480 kDa), bovine serum albumin (MW = 60 kDa), and cytochrome C (MW = 14 kDa).

### Incorporation of siRNAs into PAs Conjugated HumAfFt

A smart pool of four siRNAs targeting GAPDH (sequences reported in section 6 of the SI) was dissolved in 10 mM Tris/HCl, pH = 8 at a final concentration of 100 μM. The optimal experimental conditions for siRNA encapsulation required the addition of 20 μM siRNA mixture to a disassembled HumAfFt in 20 mM Hepes, pH = 7.4 with a molar ratio of siRNA/PA linked-HumAfFt = 5:1 at 10 °C under mild agitation. The siRNA encapsulation followed the salt-dependent assembly by adding 50 mM MgCl_2_ to promote protein association to the stable assembled form. The reassembled siRNA-PAs-HumAfFt was passed through 50 kDa centrifugal filters to remove the excess of siRNA by serial dilution. The siRNA encapsulation efficiency in PAs-HumAfFt was calculated by UV–vis spectroscopy with a Jasco V-750 (JASCO Corporation, Tokyo, Japan) as a ratio of FITC-siRNA/protein concentration by measuring FITC-siRNA absorbance at 495 nm (ε_495_ = 68 000 M^–1^ cm^–1^) and protein absorbance at 280 nm. After encapsulation, the retention of the assembled 24-meric structure was analyzed by dynamic light scattering (DLS) as described in section 5.2 of SI.

### Gel Electrophoresis and Stability of siRNA-PAs-HumAfFt after RNase Digestion

Reassembled PAs-HumAfFt with siRNA were loaded in a GreenSafe DNA stain (Canvax) containing 2% agarose gel before and after treatment with RNase A (0.5 μg/μL in 10 mM Tris/HCl pH 8.0) at 37 °C for 30 min. An identical amount of naked siRNA was used as control. The siRNA in agarose gel was visualized by using the fluorescent dye GreenSafe DNA stain (Canvax) with a Gel Doc imager. The quantification of the signal intensity was carried out with the ImageJ software.

### Cell Cultures and Transfection

Human hepatocellular carcinoma HepG2, human breast adenocarcinoma MCF-7, and human cervix adenocarcinoma HeLa cells were cultured in DMEM supplemented with 10% fetal bovine serum (FBS) and 1% penicillin/streptomycin. Transfection of GFP (Green Fluorescent Protein) expression vector together with 100 nM siRNA-GAPDH or 100 nM siRNA negative control (Sigma) was performed with the TransIT-TKO/TransIT-LT1 Transfection Reagent (Mirus) per manufacturer’s instruction; cells were harvested 24 h post-transfection. Transfection efficiency was evaluated by GFP expression level. Alternatively, cells were left untreated or treated with different substrates as indicated in Figure 3 at the final concentration of 300 μg mL^–1^ (600 nM in 24-meric conformation) and harvested 24 h post-treatment.

### Cell Viability Assay

HeLa cells were plated in a 96 well dish and treated for 24 h with different **PAs** at different final concentrations (100–300–500–1000 μg/mL) or with Doxorubicin 2 μM as a positive control. Assessment of cytotoxicity was performed by adding CellTiter 96 AQueous One Solution Reagent (Promega#G3582) directly to culture, incubating for 1–4 h, and then recording absorbance at 490 nm with a 96-well plate reader, according to the manufacturer’s instruction. The quantity of formazan product as measured by the amount of 490 nm absorbance is directly proportional to the number of living cells in culture. Triplicate set of experimental wells and control wells (without cells) containing the same volumes of culture medium and CellTiter 96 AQueous One Solution Reagent were prepared. The average 490 nm absorbance from the “no cell” control wells was subtracted from all other absorbance values to yield corrected absorbances.

### Cellular Uptake of siRNA-PAs-HumAfFt Nanoparticles

For flow cytometry analysis, HeLa cells were seeded on multiwell plates. Cells were incubated with **siRNA-PAs-HumAfFt** nanoparticles, prepared as described before, but using a smart pool of FITC-5′-siRNA whose sequence is shown in SI. HeLa cells were washed two times with PBS, detached with Trypsin-EDTA (Euroclone), washed with PBS, and resuspended in BD-FACS Flow buffer. Control cells were treated in the same way but without **PAs-HumAfFt** incubation. Internalization of conjugated nanoparticles before and after TB treatments was measured at the BD LSRFORTESSA (BD Biosciences, San Jose, CA, USA) equipped with a 488 nm laser and FACSDiva software (BD Biosciences version 6.1.3). Live cells were first gated by forward and side scatter area (FSC-A and SSC-A) plot, and then detected in the green channel for FITC expression (530/30 nm filter) and side scatter parameter. The gate for the final detection was set in the control sample. Data were analyzed using FlowJo 9.3.4 software (Tree Star, Ashland, OR, USA).

### RNA Extraction and Analysis

Total RNAs were isolated using E.Z.N.A. total RNA Kit I (OMEGA R6834–02). cDNA was synthesized using PrimeScript RT reagent Kit (Takara Cat. # RR037A) and analyzed with gene specific primers by qPCR using the fluorescent dye SYBR Green (Biorad) in a LightCycler 480 instrument (Roche Diagnostics). β-Actin was used as internal control for normalizing equal loading of the samples. Relative expression was calculated using the comparative *C*_t_ method (2^–Δ*C*^_^t^_, Δ*C*_t_ = *C*_t_(GAPDH) – *C*_t_(β-actin).

### Immunoblotting

Cells were lysed in RIPA buffer (25 mM Tris–HCl pH 7.4, 150 mM NaCl, 1 mM EDTA, 1% NP-40, sodium deoxycholate 0.5%, SDS 0.1%). Samples were analyzed by electrophoresis with Bis–Tris minigels (NuPAGE, Inc.) and immunoblotted with the following antibodies: anti-Actin (sc-1616) from Santa Cruz Biotechnology; anticleaved-PARP(#9541) and anticleaved-Caspase9 (#9505) from Cell Signaling. Proteins of interest were detected with HRP-conjugated anti-mouse/rabbit/goat IgG antibodies from Santa Cruz Biotechnology and visualized with the ECL Western blotting substrate (BioRad), according to the provided protocol. Images were captured with a ChemiDoc XRS+(Bio-Rad) imaging system.

### Statistics

P-values were determined using the one-tailed Student’s *t* test: *0.01 ≤ *P* < 0.05; **0.001 ≤ *P* < 0.01; ***P < 0.001. Results are expressed as a mean of three independent experiments bars indicating Standard Error.

### *Ab Initio* p*K*_a_ Calculations

The protonation state of tested polyamines was assessed by *ab initio* calculations. Compounds were sketched in three-dimensional format by Maestro, Release 2019–1 (Schrodinger, LCC, New York, NY), while p*K*_a_ computation was carried out with Jaguar p*K*_a_, generating up to 5 conformations for each species in water solvent, with an energy window of 12 kcal/mol and using the thorough accuracy setting.

## Supporting Information Available

NMR spectra; crystal data; p*K*_a_ values of polyamine-thiol-reactive linkers; protein mass spectrometry; size exclusion chromatography analysis; siRNA synthesis; DLS measurements; FACS analysis (PDF)
