# Supplementary material for: Design and Synthesis of Piperazine-Based Compounds Conjugated to Humanized Ferritin as Delivery System of siRNA in Cancer Cells
Source: Bioconjug Chem. 2021 May 12;32(6):1105–16. doi: 10.1021/acs.bioconjchem.1c00137 (PMC8253483; doi:10.1021/acs.bioconjchem.1c00137)
Supplement: Supplementary file 1 — bc1c00137_si_001.pdf [file bc1c00137_si_001.pdf]

Supporting Information

Design and synthesis of piperazine-based compounds conjugated to Humanized ferritin as delivery system of siRNA in cancer cells

Natalia Pediconi,[#][a] *Francesca Ghirga,*[#][a] *Cristina Del Plato,*[a],[b] *Giovanna Peruzzi,*[a] *Constantinos M. Athanassopoulos,*[c],[d] *Mattia Mori,*[e] *Maria Elisa Crestoni,*[b] *Davide Corinti,* [b] *Franco Ugozzoli,*[f] *Chiara Massera,*[g] *Alessandro Arcovito,*[h],[i] *Bruno Botta,**[b] *Alberto Boffi,*[a],[d],[j] *Deborah Quaglio,**[b] *and Paola Baiocco*[a],[d]

---

[a] Dr. N. Pediconi, Dr. G. Peruzzi, C. Del Plato, Prof. A. Boffi, Dr. P. Baiocco, Center for Life Nano- & Neuro-Science, Fondazione Istituto Italiano di Tecnologia (IIT), 00161 Rome, Italy

[b] Dr. F. Ghirga, C. Del Plato, Prof. M.E. Crestoni, Dr. D. Corinti, Prof. B. Botta, Dr. D. Quaglio, Department of Chemistry and Technology of Drugs, "Department of Excellence 2018−2022", Sapienza University of Rome, P.le Aldo Moro 5, 00185 Rome, Italy; E-mail:deborah.quaglio@uniroma1.it, bruno.botta@uniroma1.it

[c] Prof. C.M. Athanassopoulos, Department of Chemistry, University of Patras, GR-26504 Rio - Patras, Greece

[d] Prof. C.M. Athanassopoulos, Prof. A. Boffi, Dr. P. Baiocco, Department of Biochemical Sciences "Alessandro Rossi Fanelli", Sapienza University of Rome, P.le A. Moro 5, 00185 Rome, Italy

[e] Dr. M. Mori, Department of Biotechnology, Chemistry and Pharmacy, "Department of Excellence 2018−2022", University of Siena, via Aldo Moro 2, 53100, Siena, Italy

[f] Prof. F. Ugozzoli, Department of Engineering and Architecture, University of Parma, Parco Area delle Scienze 181/A, 43124 Parma, Italy

[g] Prof. C. Massera, Department of Chemical Sciences, Life and Environmental Sustainability, University of Parma, Parco Area delle Scienze 17/A, 43124 Parma, Italy

[h] Prof. A. Arcovito, Dipartimento di Scienze Biotecnologiche di base, Cliniche Intensivologiche e Perioperatorie, Università Cattolica del Sacro Cuore , Largo F. Vito 1- 00168 - Roma, Italy

[i] Prof. A. Arcovito, Fondazione Policlinico Universitario A. Gemelli - IRCCS, Largo F. Vito 1- 00168 - Roma, Italy

[j] Prof. A. Boffi, Institute of Molecular Biology and Pathology, National Research Council, P.le A. Moro 7, 00185 Rome, Italy

# Equal contribution
*corresponding authors

Table of Contents

1. ^{1}H and ^{13}C NMR spectra 4
2. Crystal data, data collection and refinement parameters 15
3. pK_a values of polyamine-thiol-reactive linkers 18
4. Protein mass spectrometry 19
5. Assessment of HumAfFt oligomerization 20
5.1 Size Exclusion Chromatography analysis 20
5.2 Dynamic Light Scattering 21
6. siRNA sequences 21
7. TfR1 expression by Immunoblotting 22
8. FITC-siRNA-PAs-HumAfFt delivery by Flow Citometry 22
9. References 23
10. Author contributions 23

1. ^{1}H and ^{13}C NMR spectra

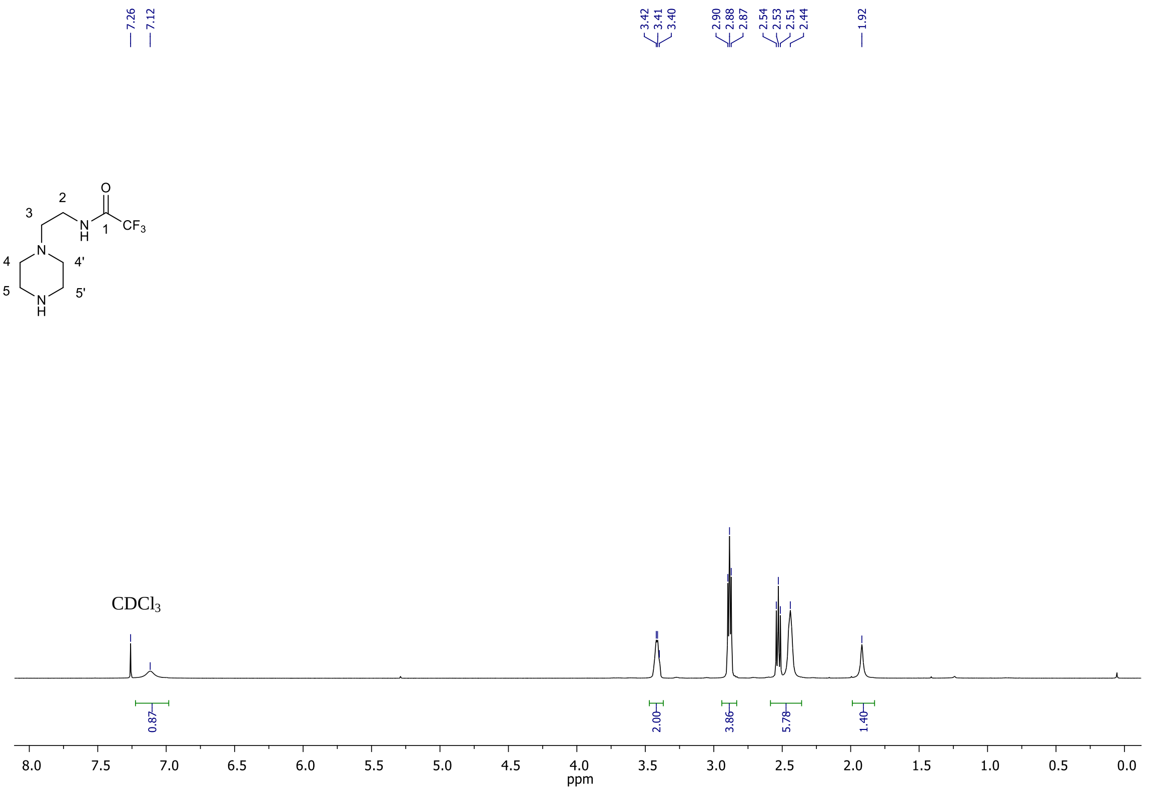

Figure S1: ^{1}H NMR ($CDCl_3$, 400 MHz) spectrum of compound **3**.

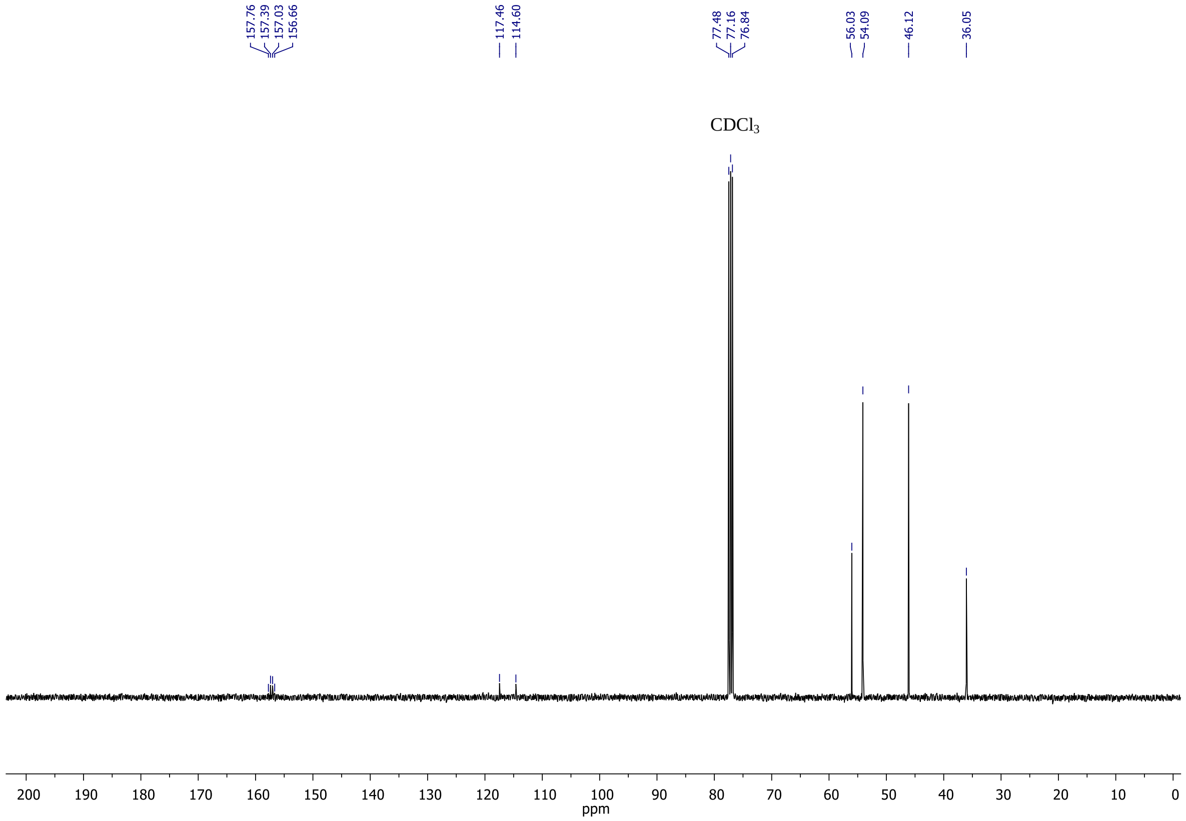

Figure S2: ^{13}C NMR ($CDCl_3$, 101 MHz) spectrum of compound **3**.

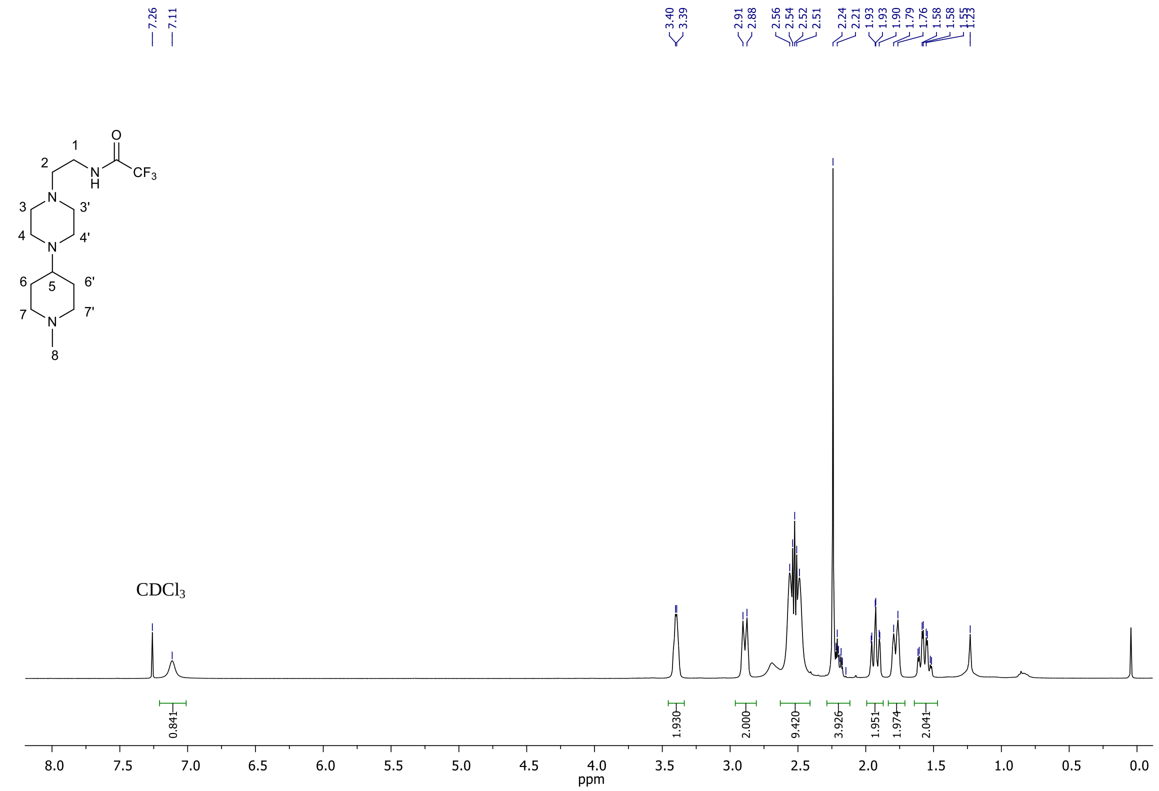

Figure S3: ^{1}H NMR ($CDCl_3$, 400 MHz) spectrum of compound **5**.

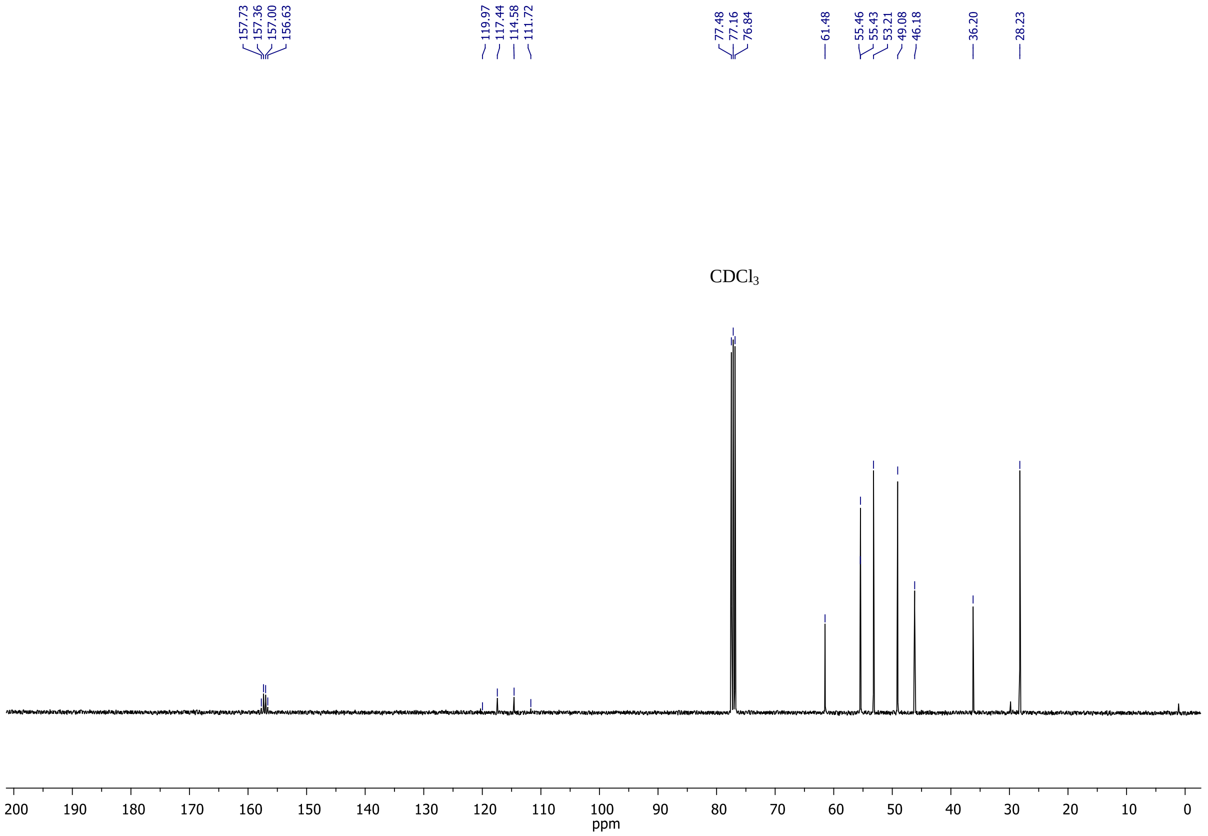

Figure S4: ^{13}C NMR ($CDCl_3$, 101 MHz) spectrum of compound **5.**

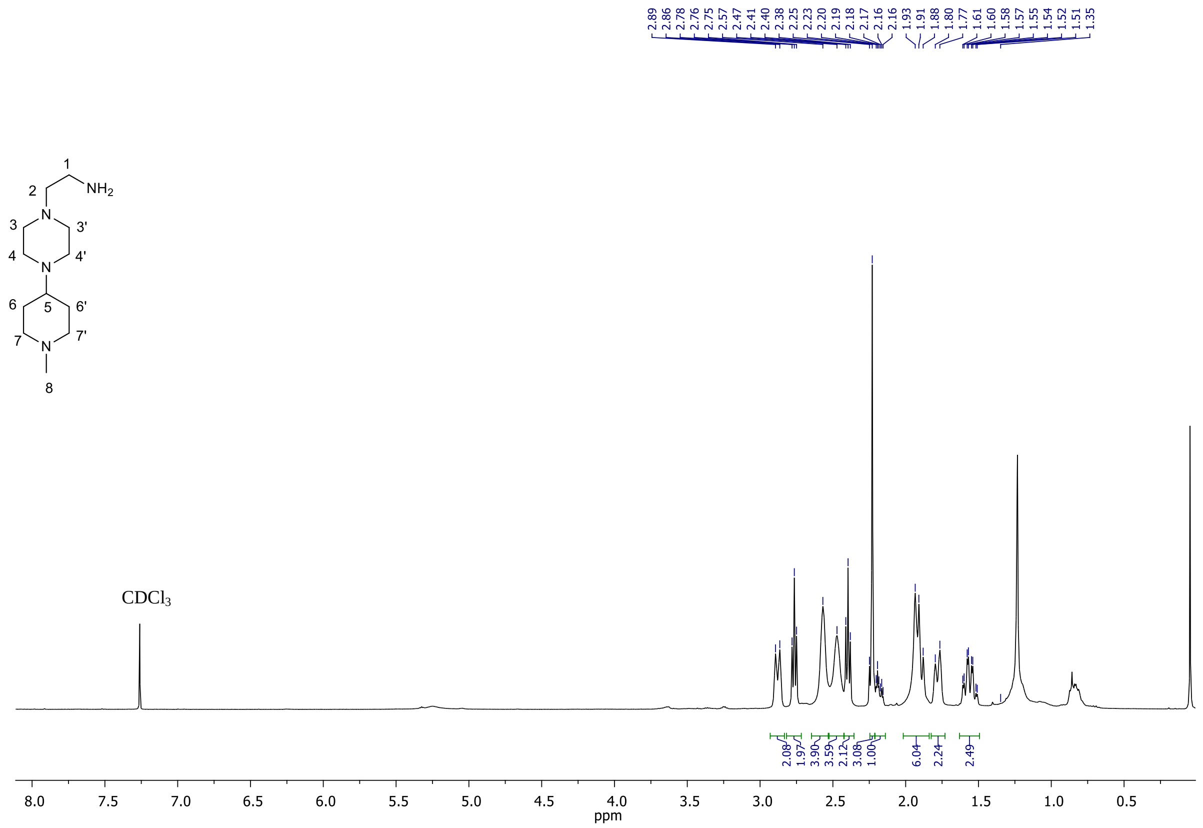

Figure S5: ^{1}H NMR ($CDCl_3$, 400 MHz) spectrum of compound **PA2**.

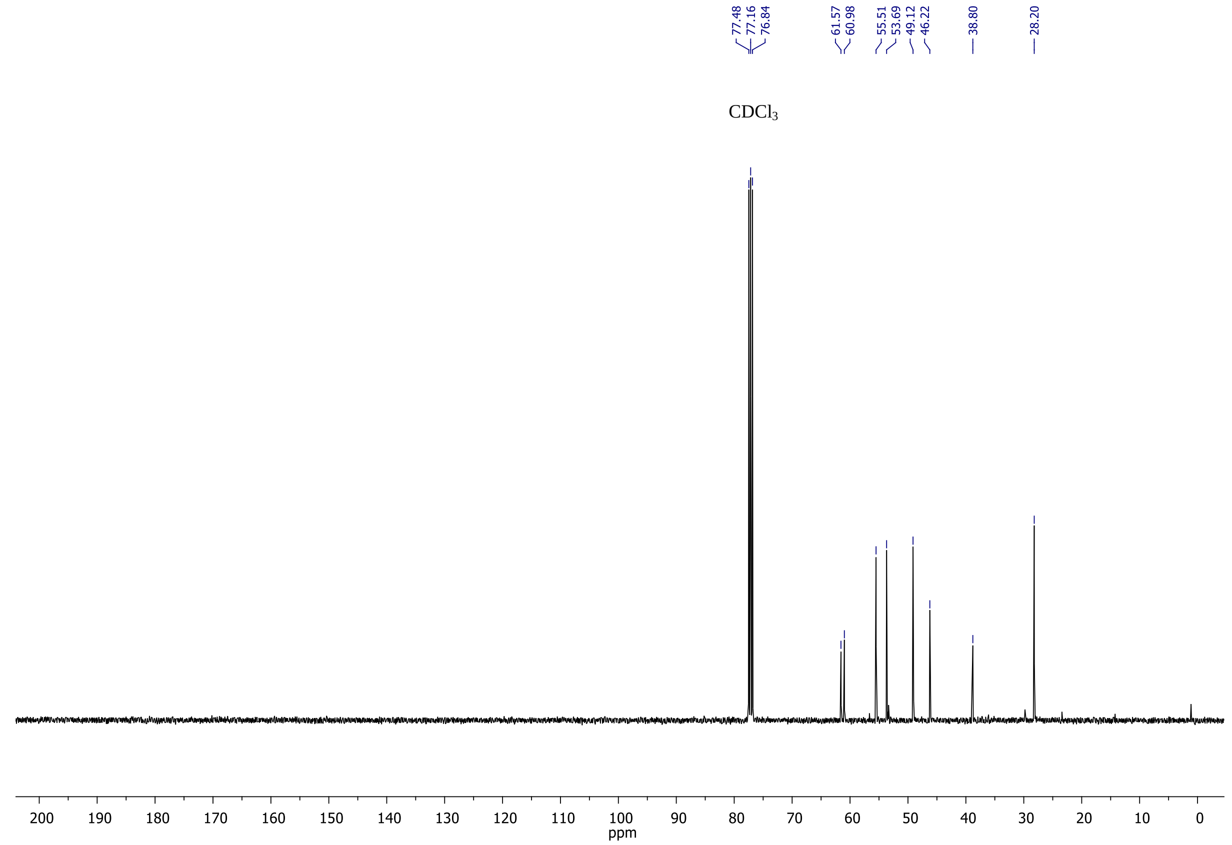

Figure S6: ^{13}C NMR ($CDCl_3$, 101 MHz) spectrum of compound **PA2**.

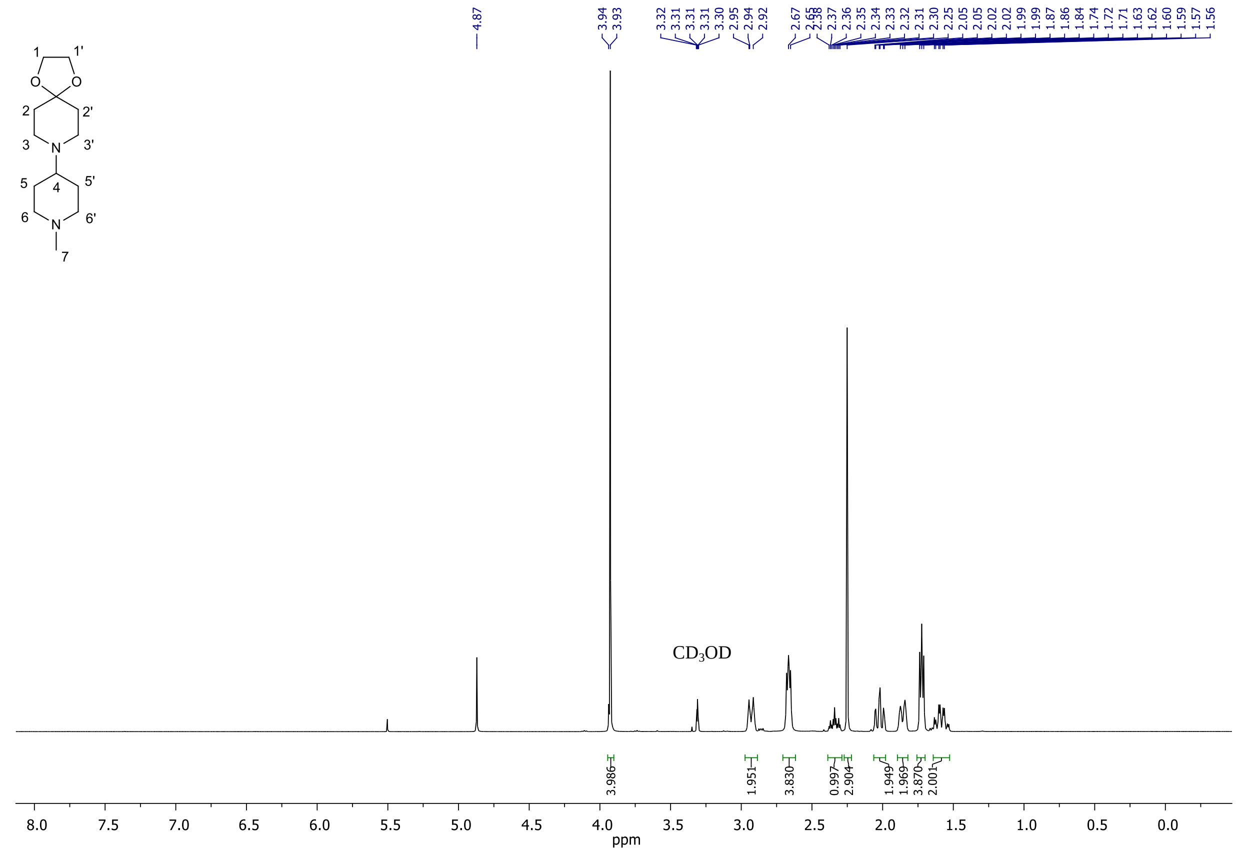

Figure S7: ^{1}H NMR (CD_3OD, 400 MHz) spectrum of compound **7**.

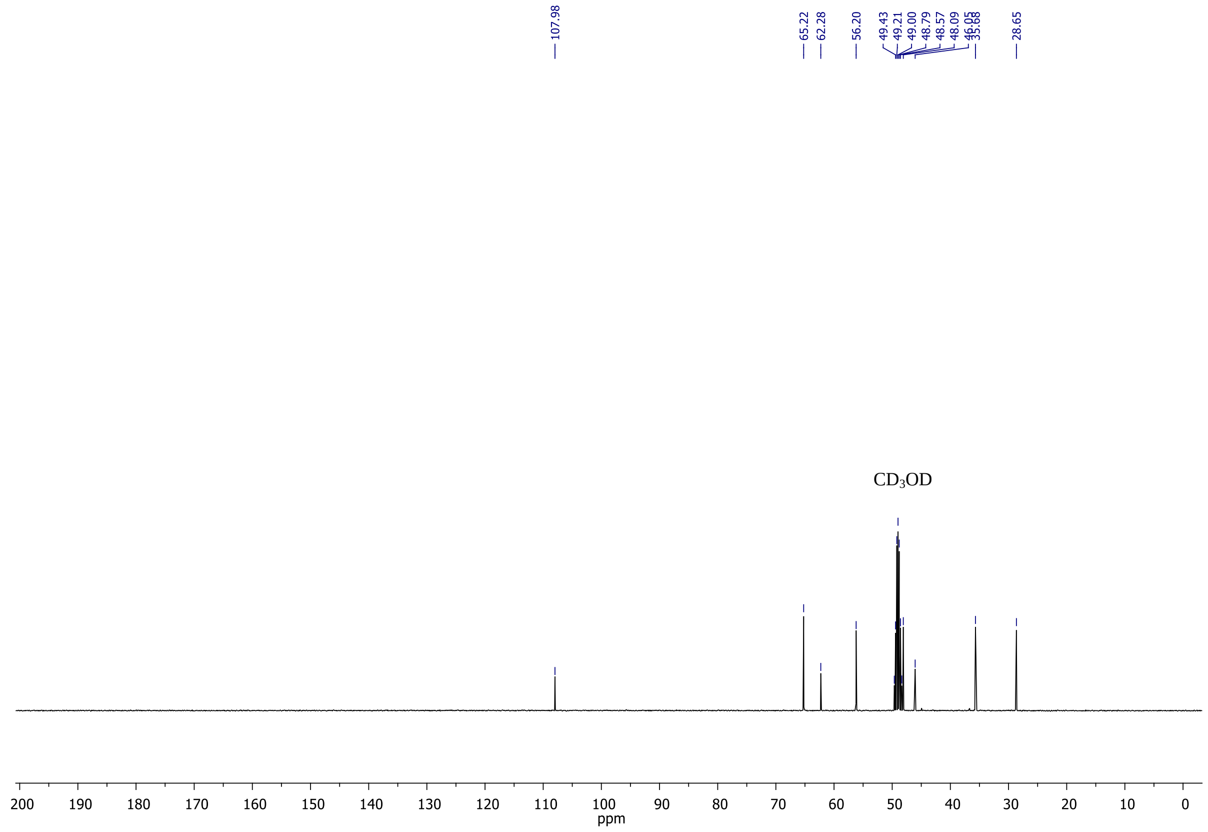

Figure S8: ^{13}C NMR (CD_3OD, 101 MHz) spectrum of compound **7**.

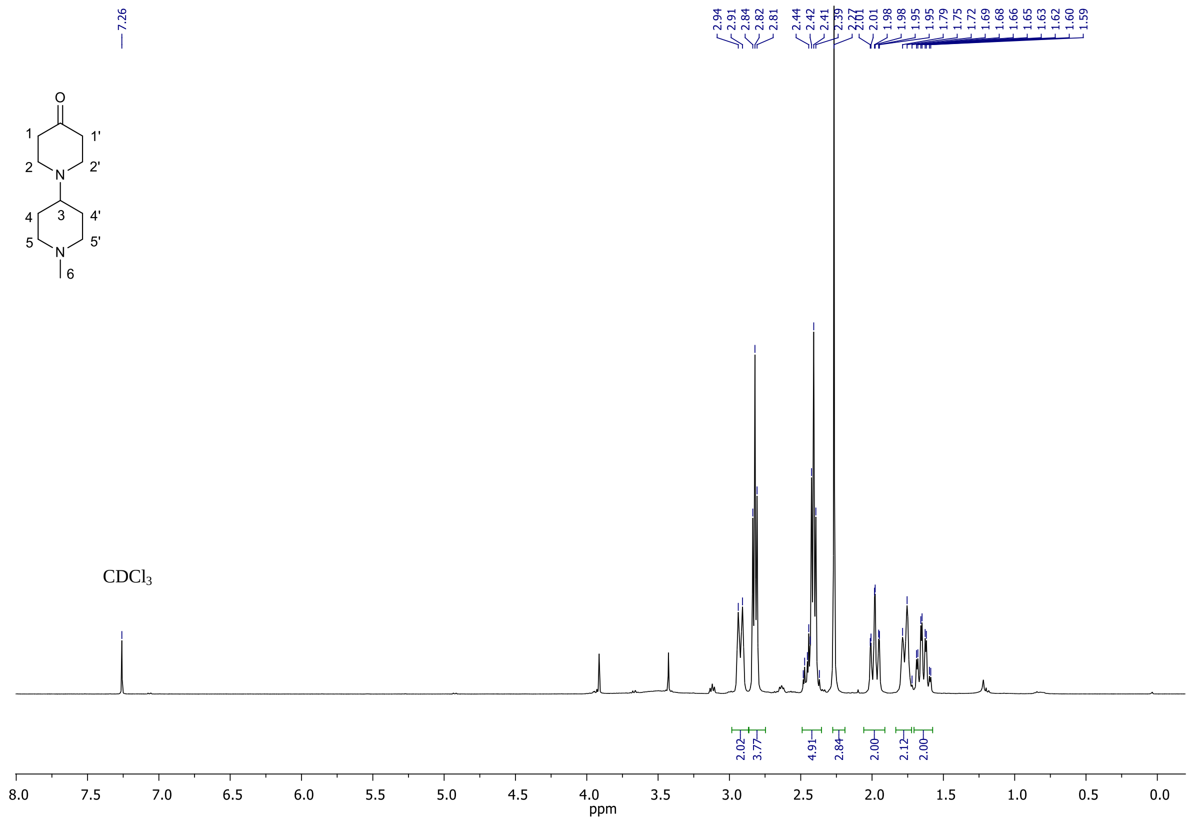

Figure S9: ^{1}H NMR ($CDCl_3$, 400 MHz) spectrum of compound **8**.

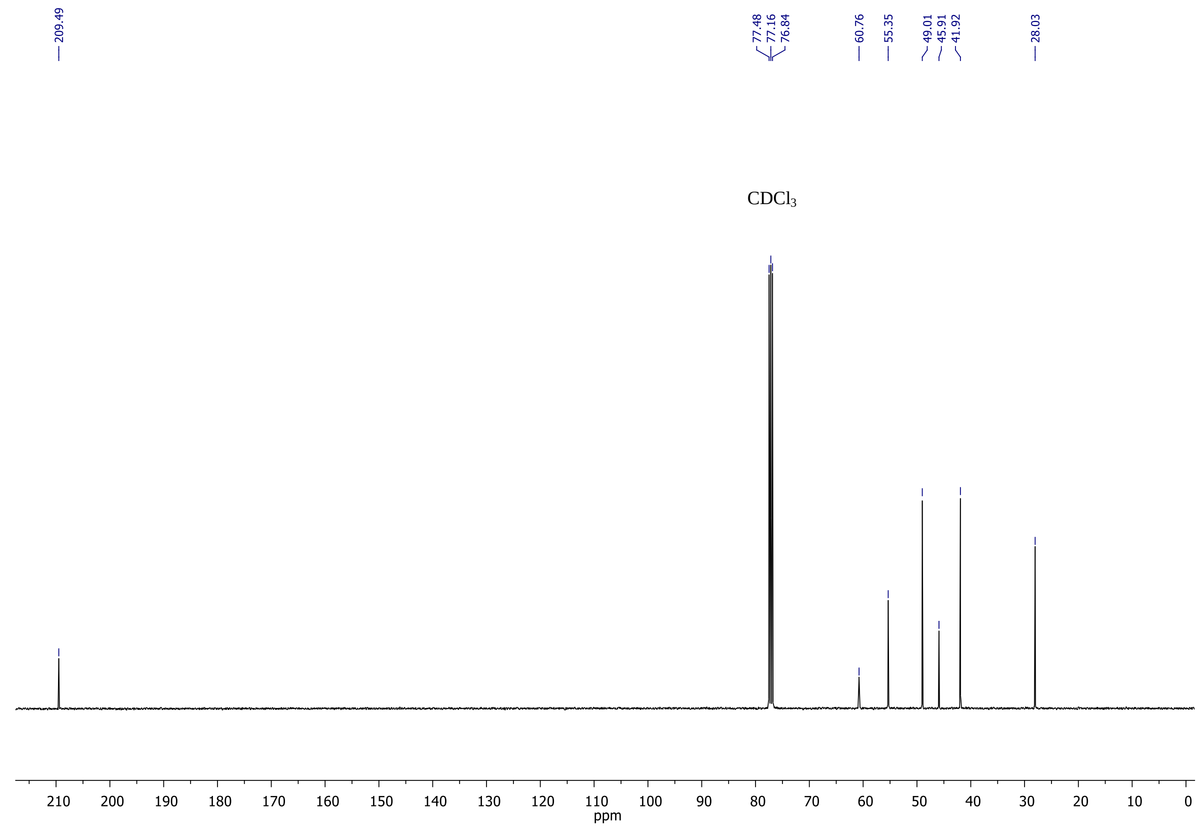

Figura S10: ^{13}C NMR ($CDCl_3$, 101 MHz) spectrum of compound **8**.

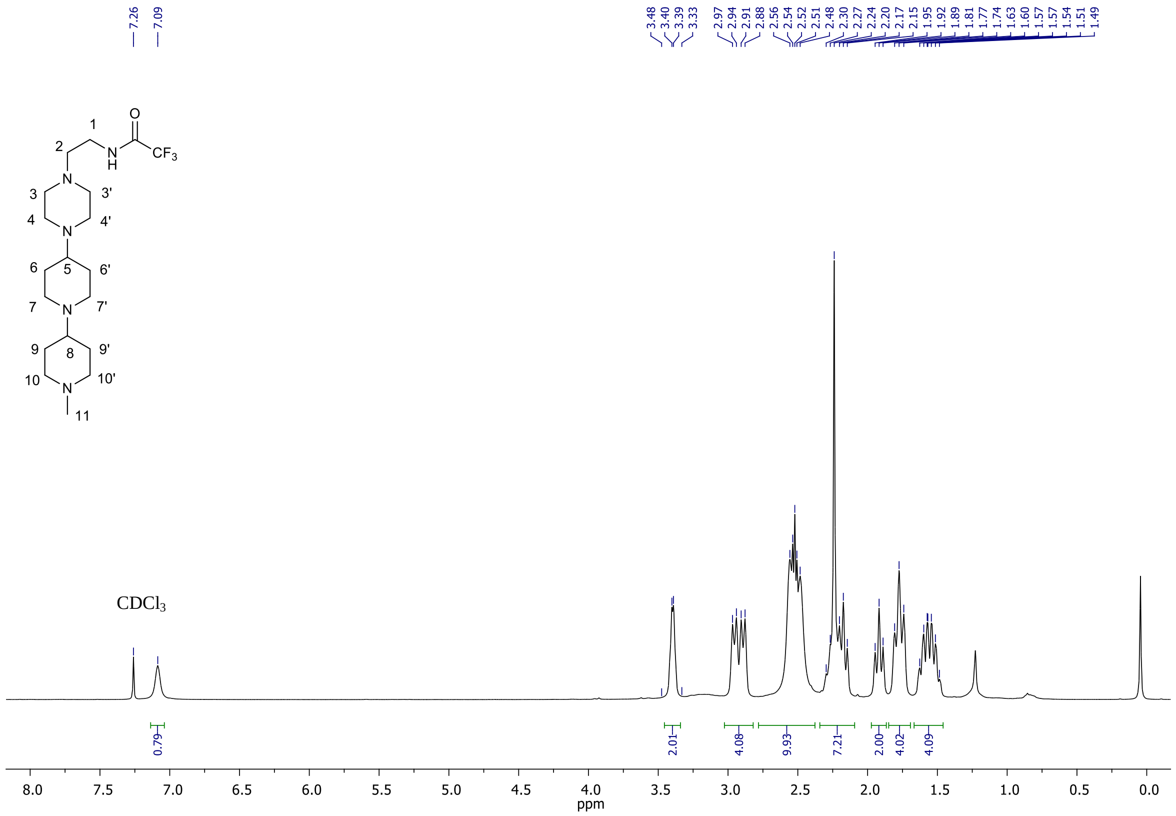

Figure S11: ^{1}H NMR ($CDCl_3$, 400 MHz) spectrum of compound **9**.

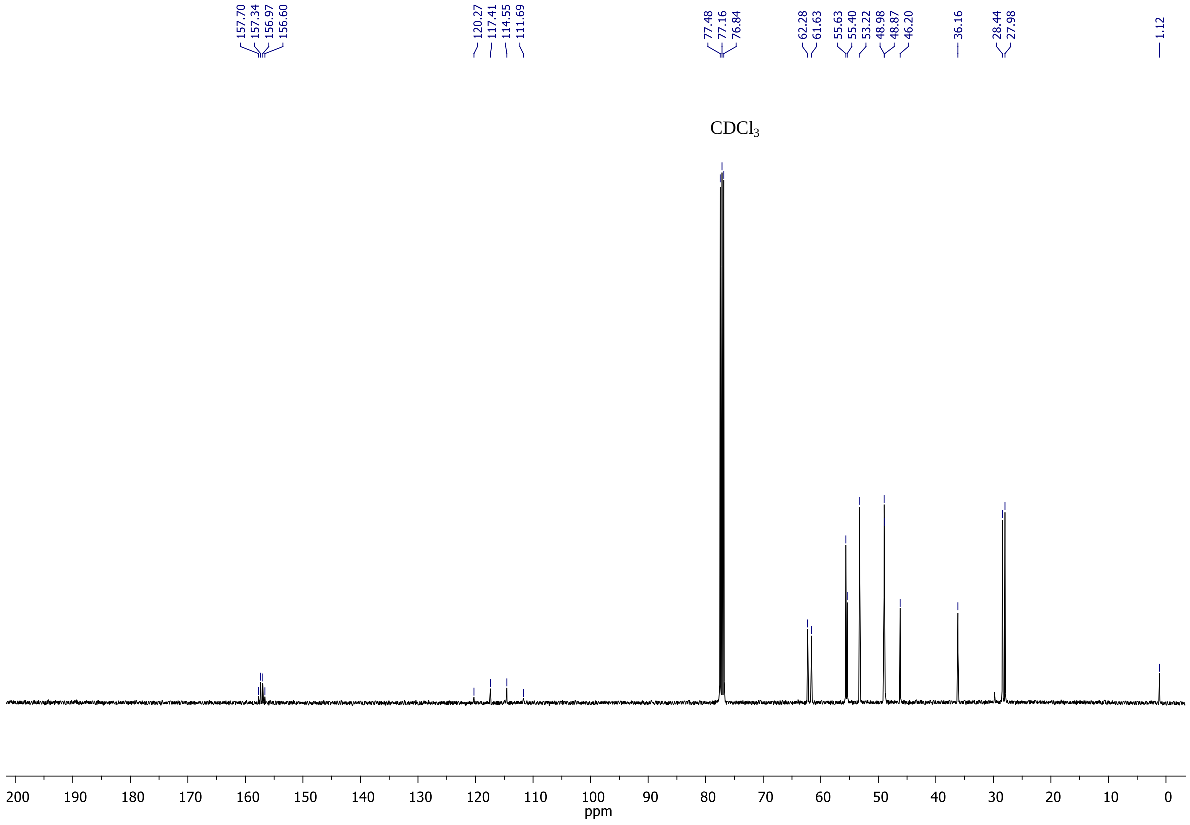

Figure S12: ^{13}C NMR ($CDCl_3$, 101 MHz) spectrum of compound **9**.

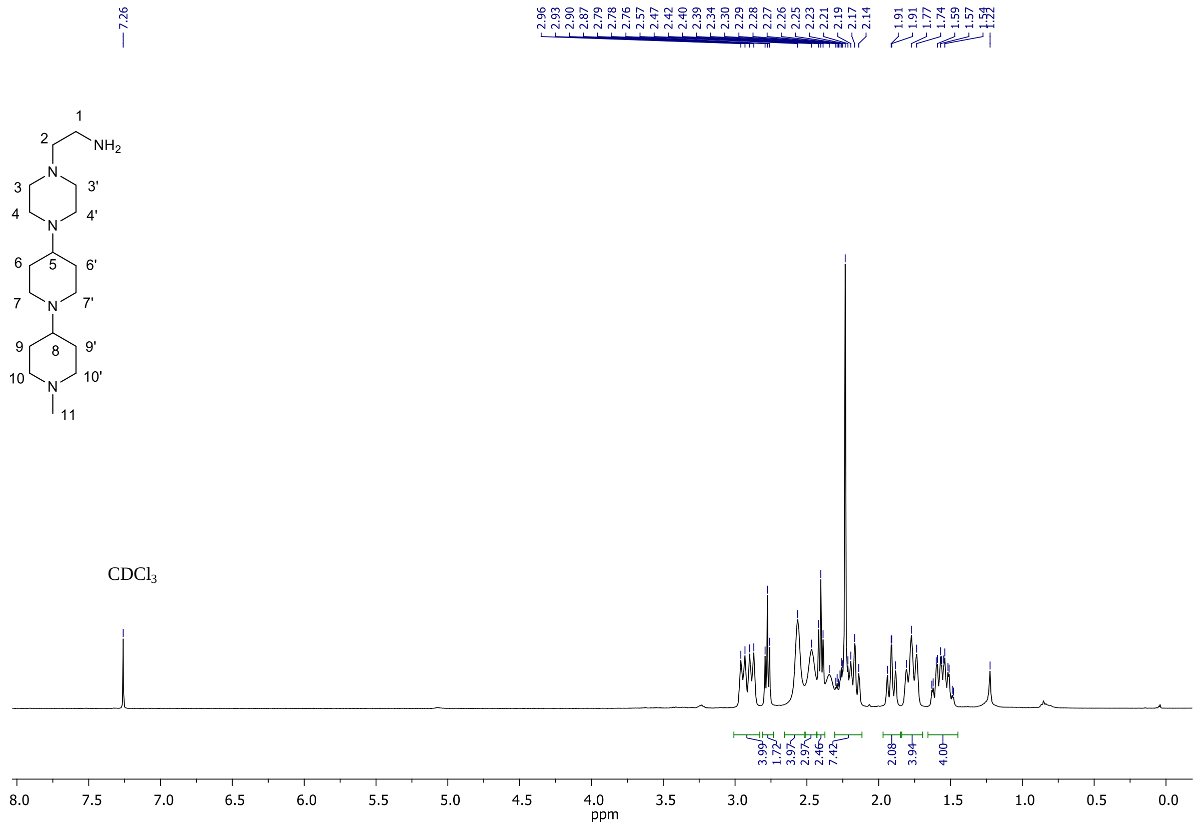

Figure S13: ^{1}H NMR ($CDCl_3$, 400 MHz) spectrum of compound **PA3**.

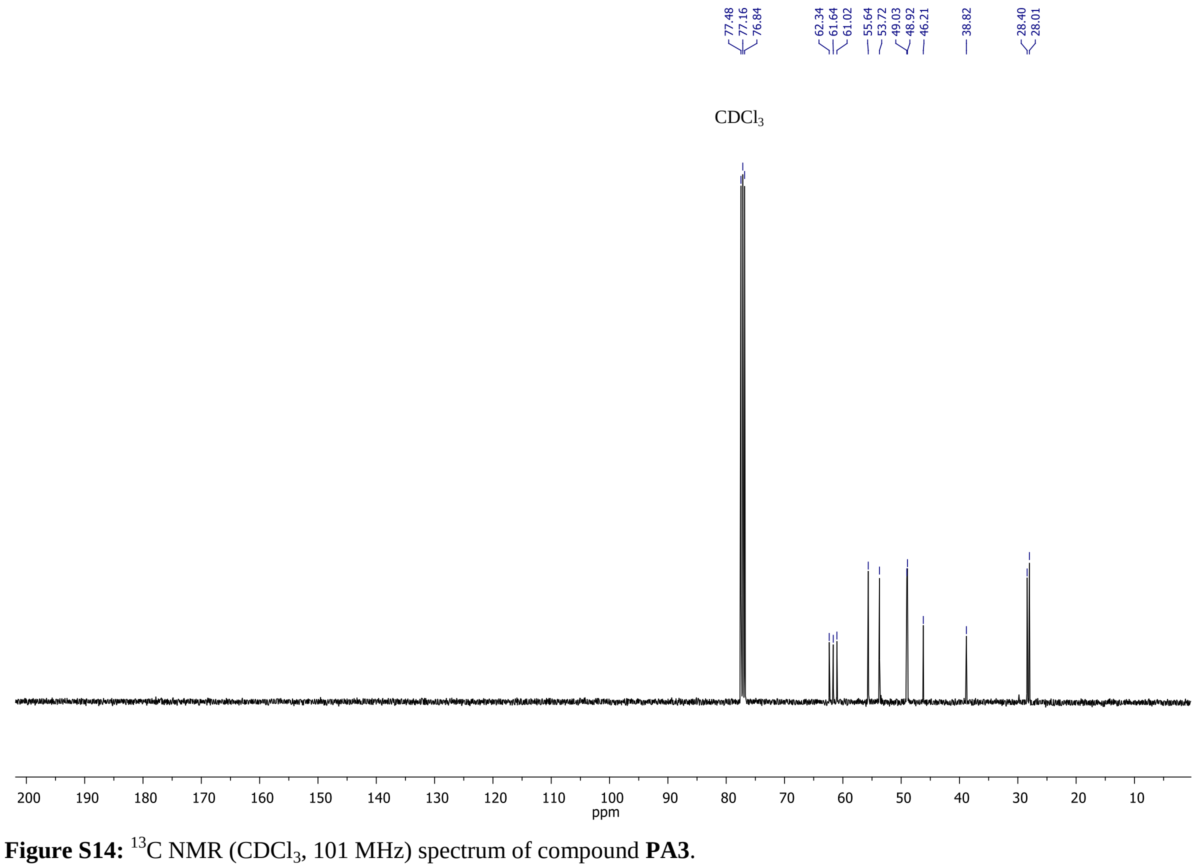

Figure S14: ^{13}C NMR ($CDCl_3$, 101 MHz) spectrum of compound **PA3**.

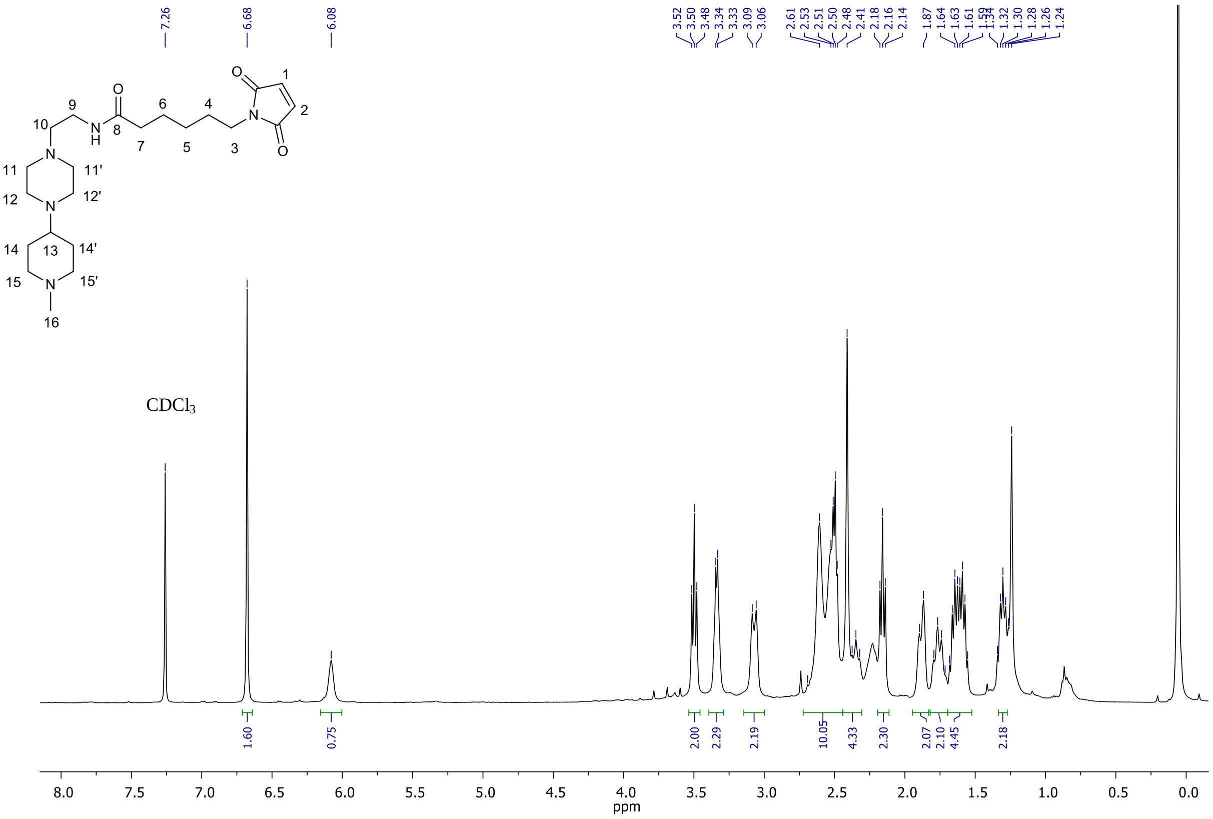

Figure S15: ^{1}H NMR ($CDCl_3$, 400 MHz) spectrum of compound **PA2.1**.

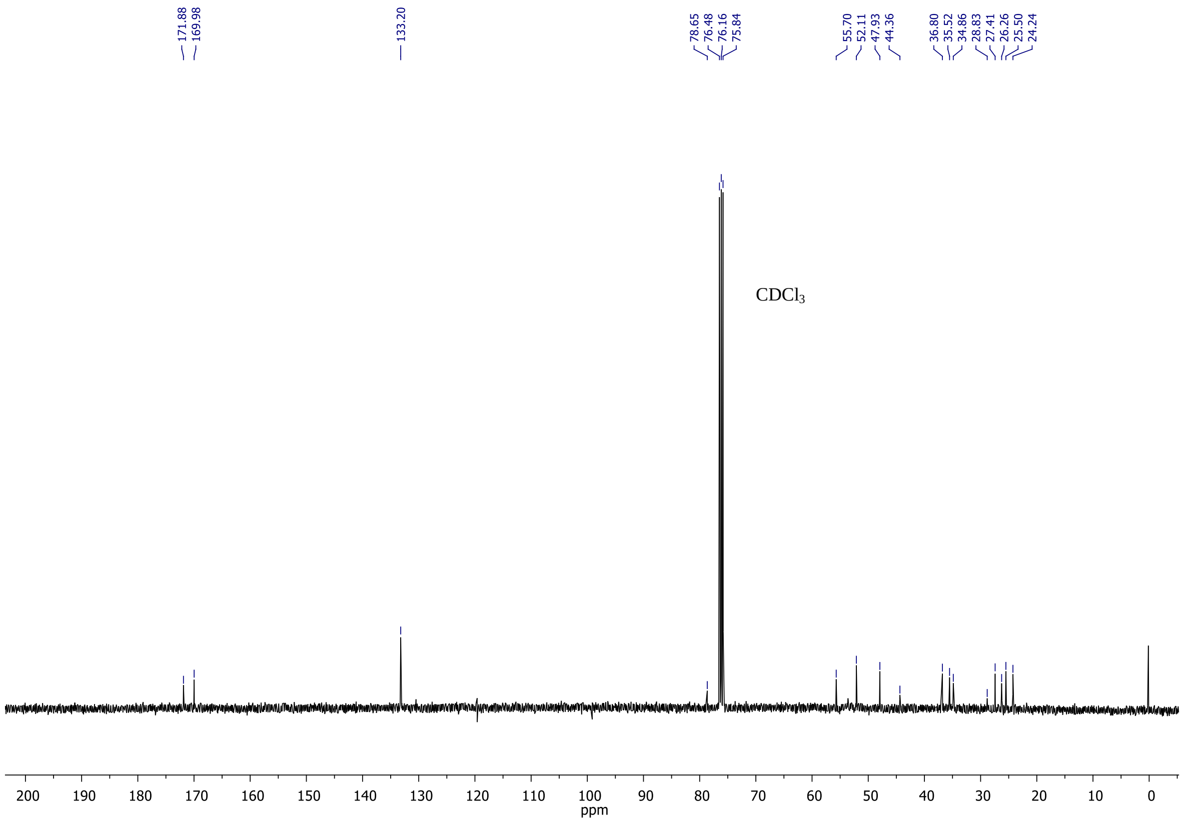

Figure S16: ^{13}C NMR ($CDCl_3$, 101 MHz) spectrum of compound **PA2.1**.

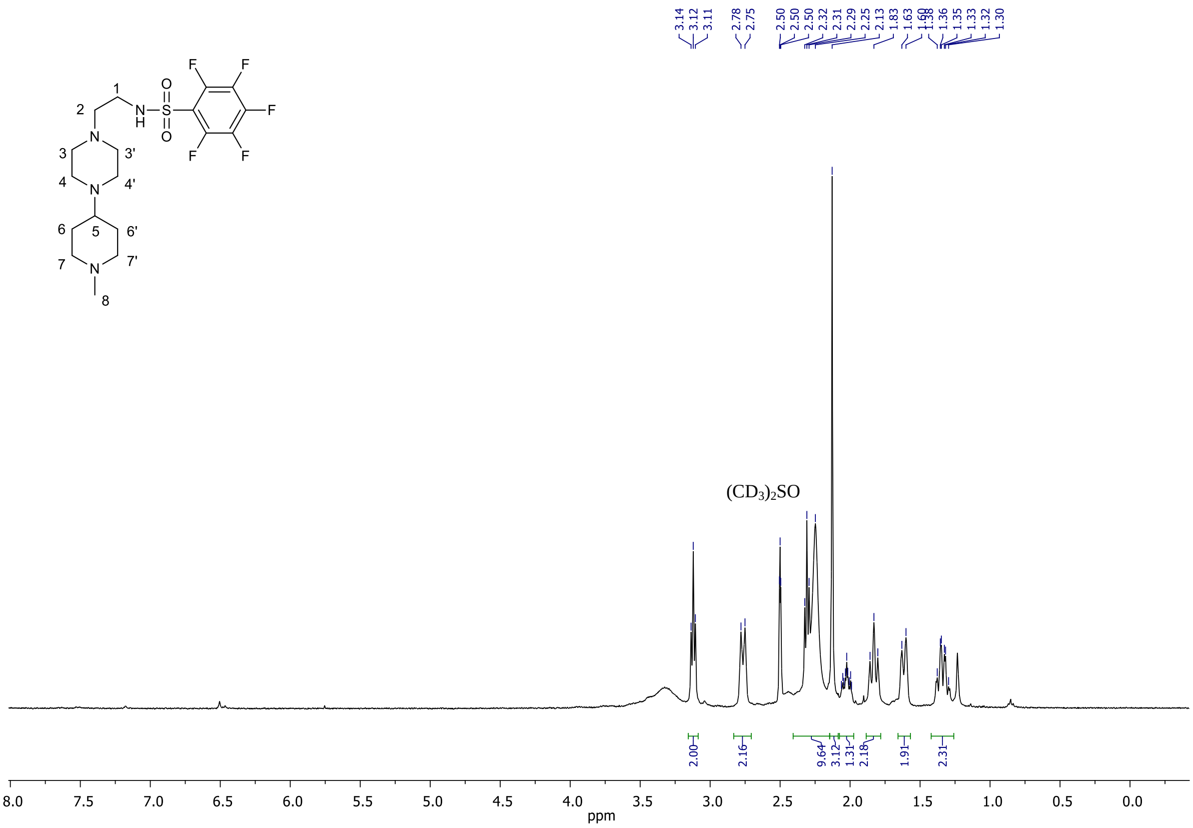

Figure S17: ^{1}H NMR ($(CD_3)_2SO$, 400 MHz) spectrum of compound **PA2.2**.

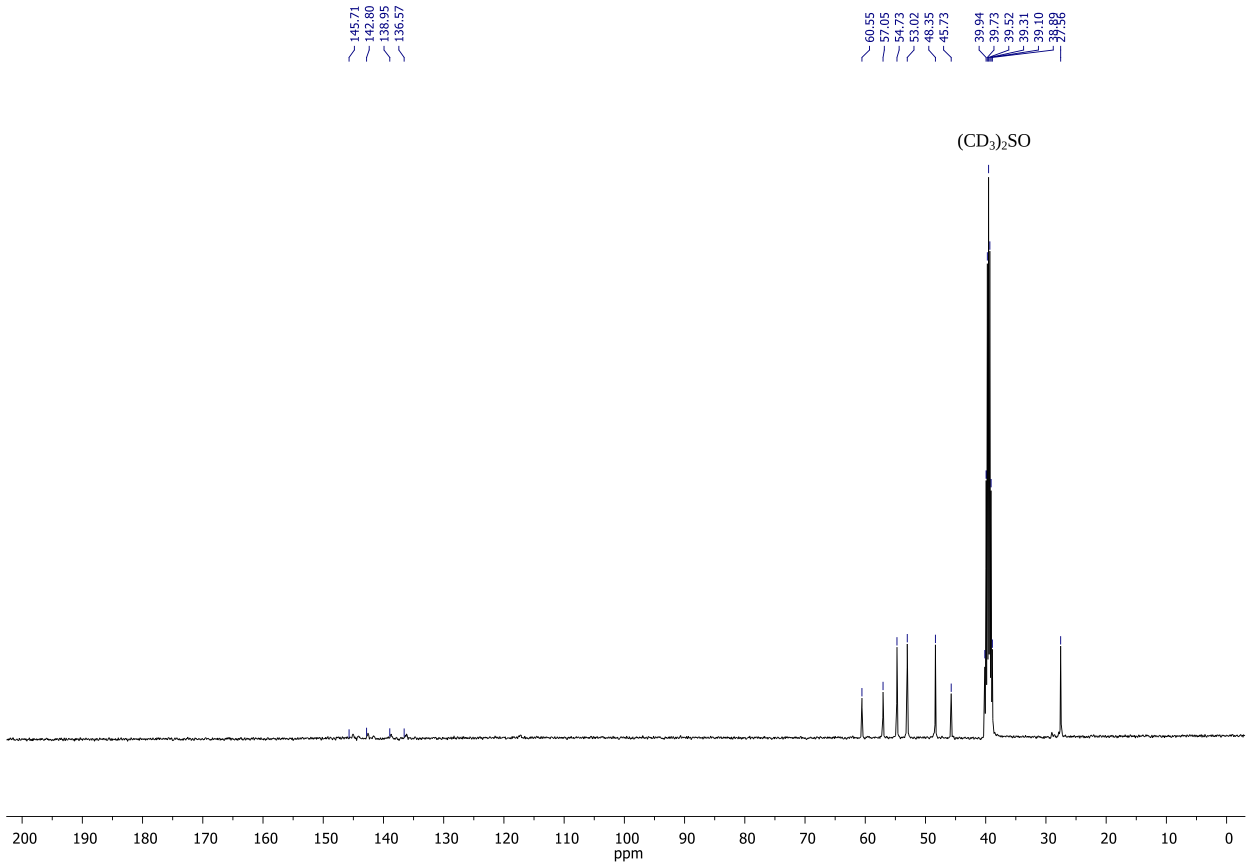

Figure S18: ^{13}C NMR ($(CD_3)_2SO$, 101 MHz) spectrum of compound **PA2.2.**

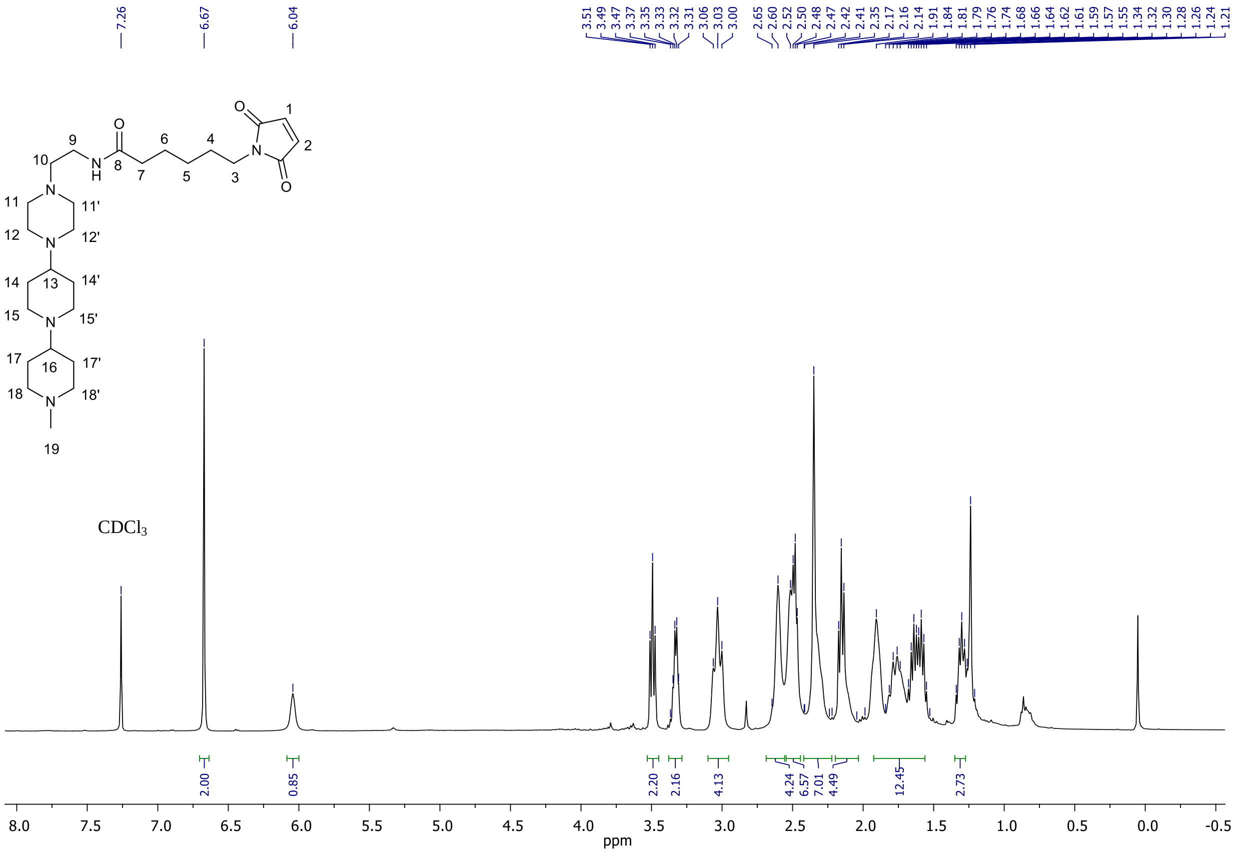

Figure S19: ^{1}H NMR ($CDCl_3$, 400 MHz) spectrum of compound **PA3.1**.

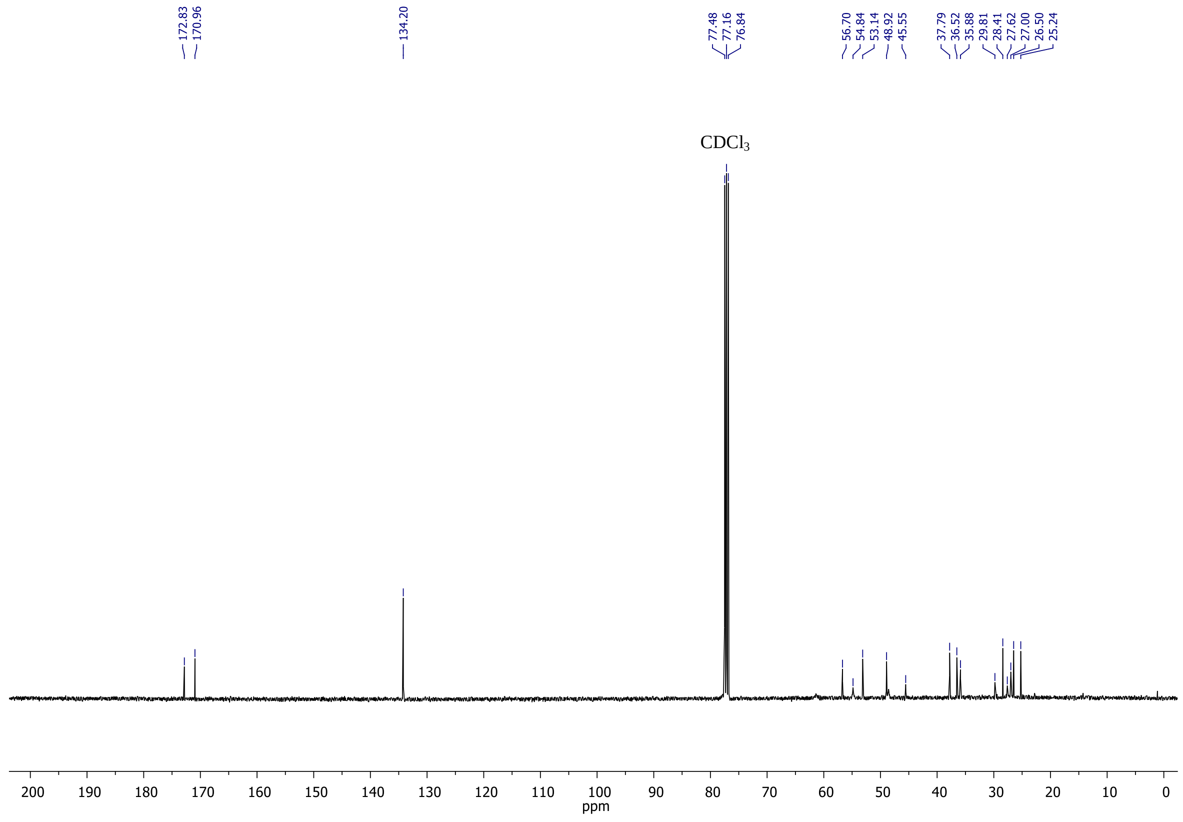

Figure S20: ^{13}C NMR ($CDCl_3$, 101 MHz) spectrum of compound **PA3.1**.

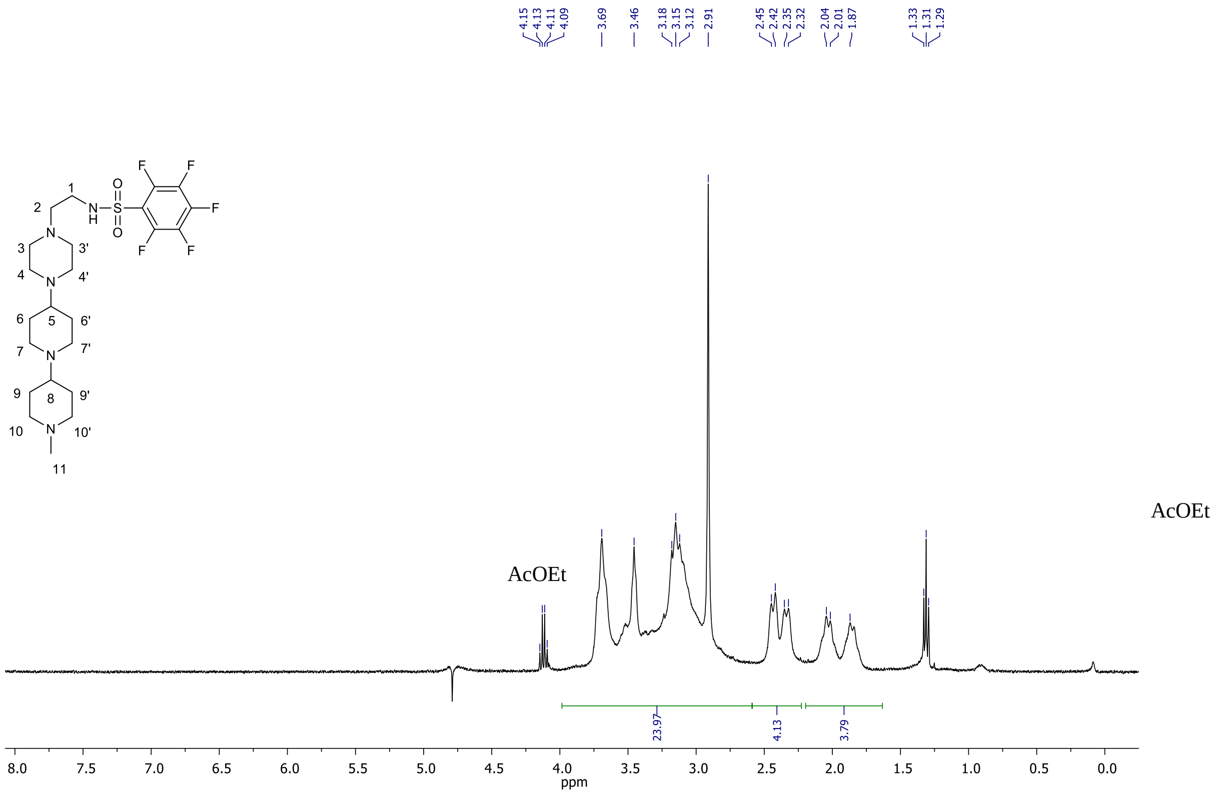

Figure S21: ^{1}H NMR (D_2O, 400 MHz) spectrum of compound **PA3.2.**

.

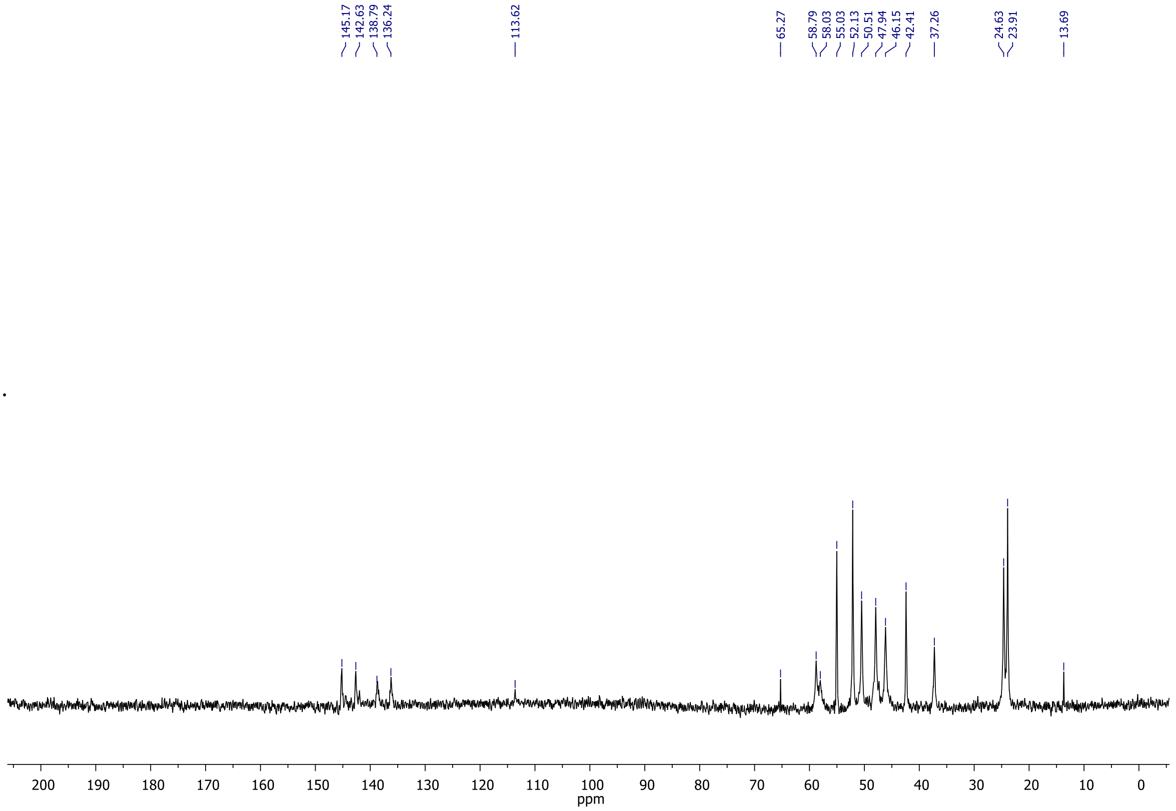

Figure S22: ^{13}C NMR (D_2O, 101 MHz) spectrum of compound **PA3.2**.

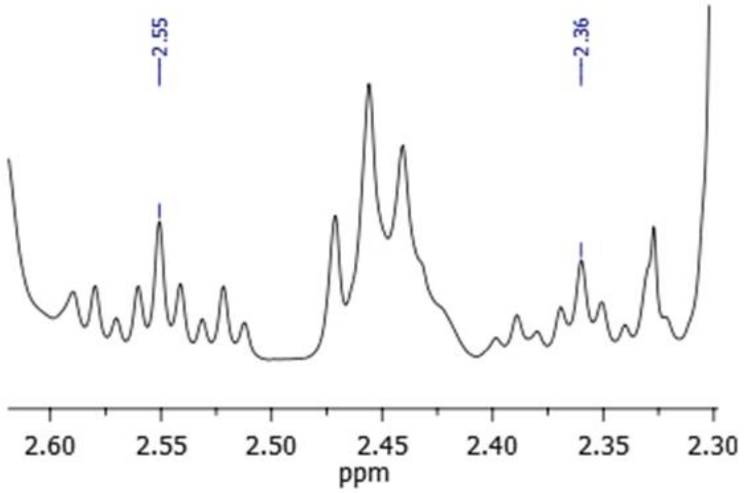

Figure S23: expansion of the ^{1}H NMR (CD_3OD, 400 MHz) spectrum of compound **PA3**.

2. Crystal data, data collection and refinement parameters

The crystal and molecular structures of polyamines **5** and **9** have been established by single crystal X-ray diffraction experiments at low temperature [200(2) K (**5**) and 170 K (**9**)] on a Bruker D8 Venture PhotonII diffractometer (Chiesi Farmaceutici SpA is acknowledged for the support with the D8 Venture X-ray equipment) equipped with a CCD detector and using a CuKα (λ = 1.54178 Å) radiation. Collected frames were processed using the SAINT and SADABS software to obtain the intensities data file.[1] All reagents were purchased from Sigma-Aldrich and were used without further purification unless stated otherwise.
Crystal data and experimental details for the X ray measurements and structure refinement are summarized in table S1.
The two structures were solved by Direct Methods using the sir97 package [2] and refined by least-squares methods on the F_o^2 by the SHELXL-2014 program [3]. All the atoms were treated with anisotropic thermal parameters with the exception of the hydrogen atoms placed at their calculated positions and refined "riding" with isotropic thermal parameters on their attached atoms [(C-H 0.98-0.99 Å, Uiso(H) = 1.2Ueq(C) and 1.5Ueq(C_{met})]. When possible, the water hydrogen atoms were located in electron density map.
In the crystal lattice of compound **9**, 5 water molecules were found; two of them (O1W e O4W) are disordered on two different positions with site occupancy factors of 0.3 and 0.7, respectively. In the case of O5W, the molecule is disordered over two positions each with occupancy of 0.5. One of the two positions could not be refined, and its contribution was taken into account using the program SQUEEZE [4]. The program calculated a solvent accessible volume of 32 $Å^3$ and 15 electron per unit cell. The weighting scheme used in the last cycle of the refinement was $w = 1/\ \sigma^2 F_o^2 + (0.1464P)^2 + 0.9437P$] e $w = 1/\ [\sigma\ ^2F_o^2 + (0.3982P)^2]$ [dove $P = (F_o^2 + 2F_c^2)/3$] for compounds **5** and **9**, respectively.
Crystallographic data have been deposited with the Cambridge Crystallographic Data Centre as supplementary publication no. CCDC-1992283 and 1992284 and can be obtained free of charge on application to the CCDC, 12 Union Road, Cambridge, CB2 1EZ, UK (http://www.ccdc.cam.ac.uk).

Chemical formula	$C_{14}H_{27}F_3N_4O_2$	$C_{19}H_{42}F_3N_5O_6$
Formula weight	340.39	493.57
crystal system	Monoclinic	Triclinic
space group	*P*21/*n*	*P*-1
a/Å	13.4089(9	6.968(1)
b/Å	9.5092(6	11.707(2)
c/Å	14.5949(9)	16.987(3)
α/°	-	104.330(8)
β/°	107.275(3)	91.491(7)
γ/°	-	99.349(7)
V/Å^3	1777.0(2)	1321.6(4)
Z	4	2
D_c/g cm^{-3}	1.269	1.240
F(000)	724	532
μ/mm^{-1}	0.916	0.896
$\theta_{min,max}$/°	3.934-75.176	2.691-58.764
Total reflections	22786	25473
Unique reflections	3633 [R(int) = 0.673]	3573[R(int) = 0.0721]
Observed reflections	2896	2129
data collection/refinement /parameters	3633 / 0 / 221	3573 / 0 / 309
S^a	0.999	1.009
R[Fo>4 ʃ(Fo)]^b	0.0726	0.1312
wR_2^b	0.2174	0.4123
$\Delta\rho_{min,max}$/e Å^{-3}	0.708, -0.525	1.289, -0.315

[a]Goodness-of-fit S = [©w(F_o^2-F_c^2)^2/ (n-p)]1/2, dove n è il numero di riflessioni e p è il numero di parametri. [b]$R_1 = \Sigma\|F_o| - |F_c\|/\Sigma|F_o|$, $wR_2 = [\Sigma[w(F_o^2\ F_c^2)^2]/\Sigma[w(F_o^2)^2]]^{1/2}$.

Table S1: Crystallographic data and experimental details for compounds **5** and **9**.

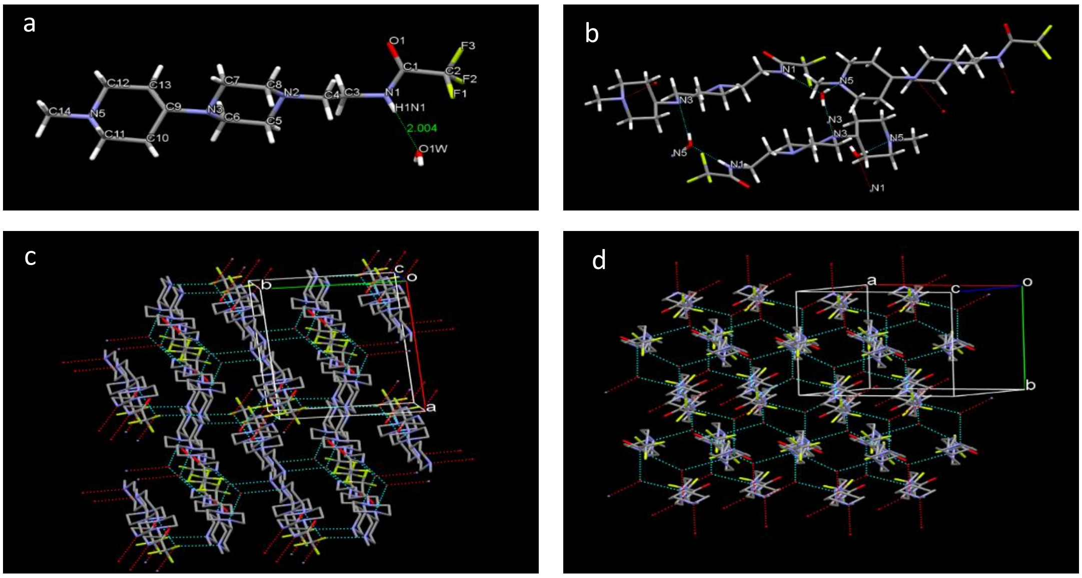

Figure S24: a) Representation of compound **5** with H_2O_{1W} responsible for the self-assembly in 3D; **b)** Self-assembly motif in the 3D network of **5** and water molecules. Each water molecule forms three hydrogen bonds linking three units of **5**: H_2O_{1W} acts as *acceptor* of the N1-H···O1W (H···O1W 2.004 Å) hydrogen bond, and contemporarily as a *donor* of the two O1W-H···N5^{i} (H···N5 1.955 Å) and O1W-H···N3^{ii} (H···N3 2.172 Å) hydrogen bonds (symmetry codes*: i = ½+x, ½-y, - ½+z; ii = 1-x, 1-y, 1-z*); **c)** Crystal structure of compound **5** viewed parallel to the a-axis direction of the unit cell with hydrogen bonds represented as light blue dotted lines. The red dotted lines show the multiple hydrogen bonds which expand the lattice; **d)** Crystal structure of compound **5** viewed parallel to the b-axis direction of the unit cell with hydrogen bonds represented as light blue dotted lines. The red dotted lines show the multiple hydrogen bonds which expand the lattice.
Colors: C, gray; N, blue; O, red; F, yellow; H, white; Hydrogen bonds are represented as light-blue dotted lines.

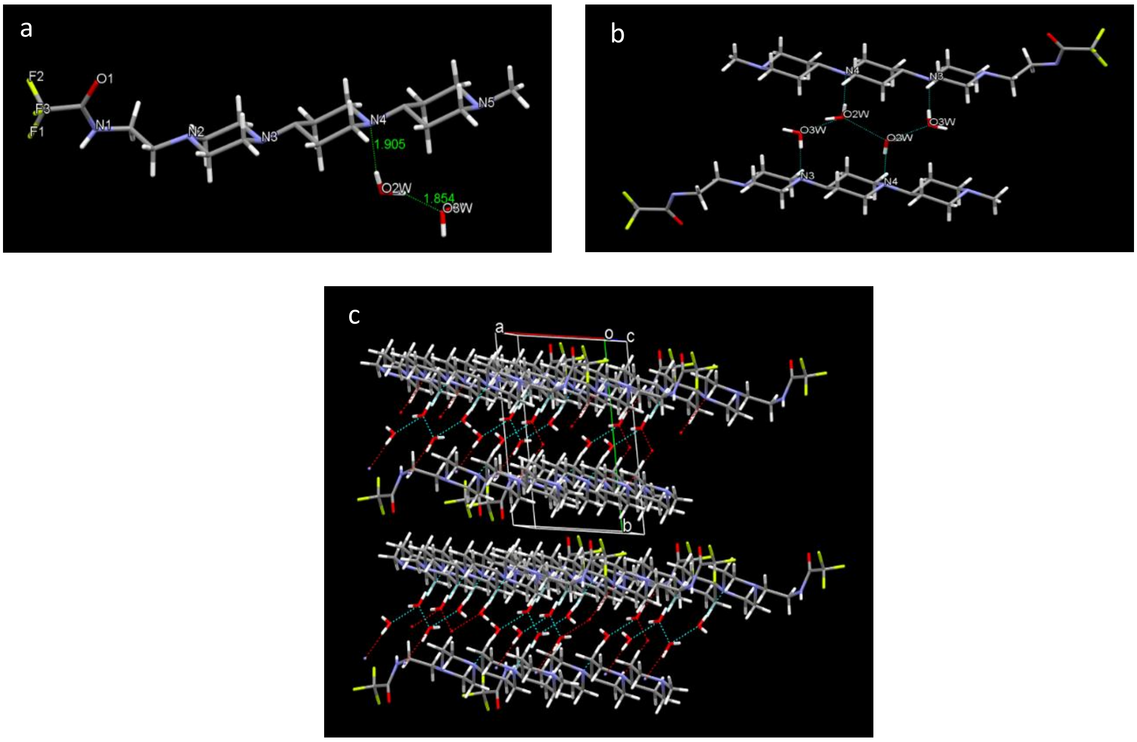

Figure S25: a) Representation of polyamine **9** with H_2O_{2W} and H_2O_{3W}: the H_2O_{2W} water molecules (which are also linked in pairs *via* hydrogen bonds) act as *donor* of the O2W-H ···N4 (H ···N4 1.905 Å) and O2W-H···O3W (H···O3W 1.854 Å) hydrogen bonds; **b)** Dimeric structure of **9** held together by multiple hydrogen bonds involving H_2O_{2W} and H_2O_{3w} water molecules (O3W-H···N3, O2W-H···N4); **c)** Perspective view of the crystal lattice in compound **9** along the **c**-axis direction of the unit cell showing the layers of dimers.
Colors: C, gray; N, blue; O, red; F, yellow; H, white; Hydrogen bonds are represented as light-blue dotted lines.

3. pK_a values of polyamine-thiol-reactive linkers

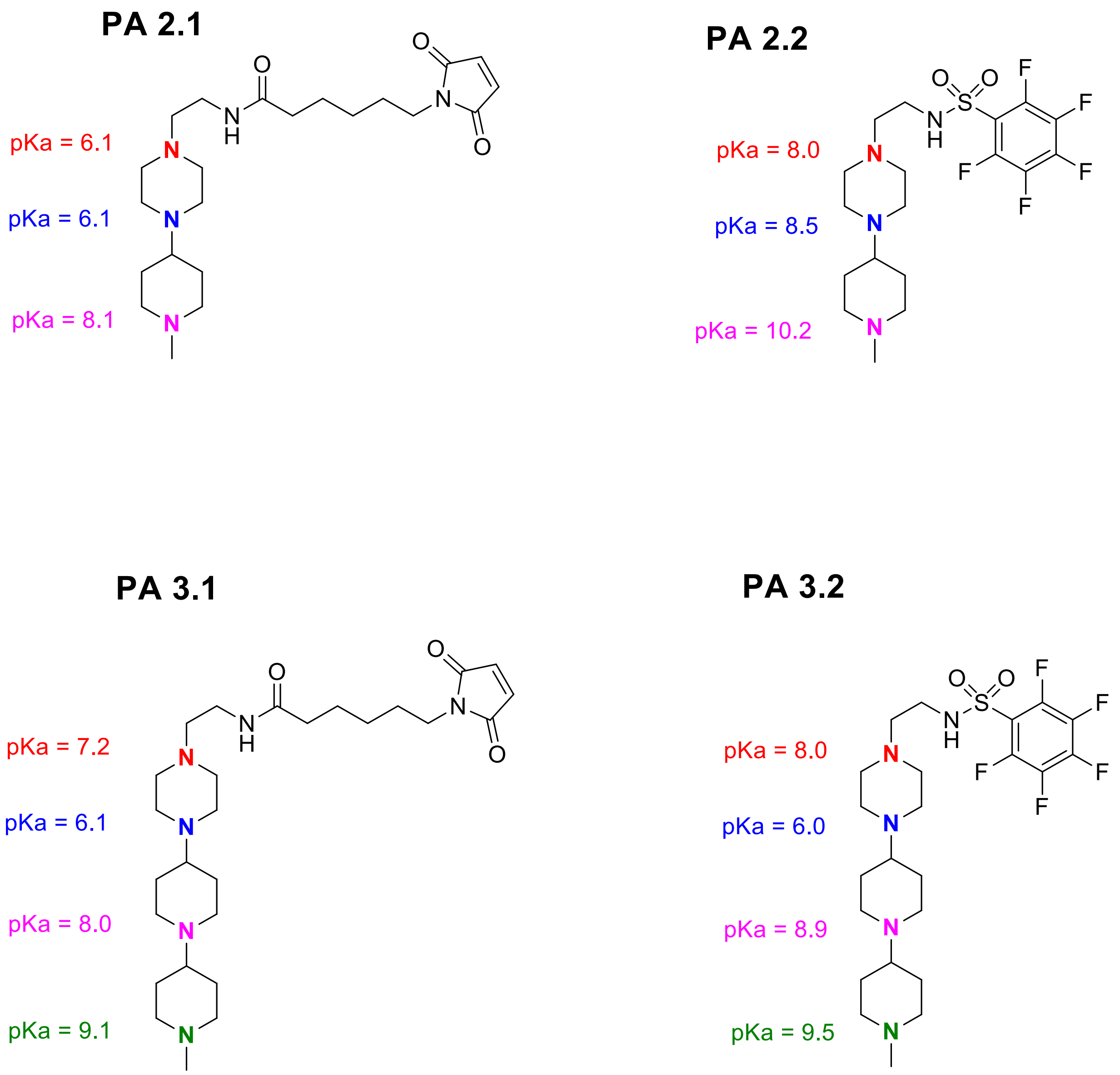

Figure S26: pK_a values of **PAs** calculated by an *ab initio* quantum chemical program (Jaguar).

4. Protein mass spectrometry

For 0.5 mL of each sample, 0.5 mL of acetonitrile and 1 µL of formic acid were added, and each solution obtained was injected into the Synapt G2 Si mass spectrometer (Waters) equipped with an electrospray ionization source, using these parameters: infusion flow rate 10 µL/min, capillary 3 kV, source temperature 120°C, sampling cone 80 V, source offset 80 V, desolvation temperature 150°C, nitrogen desolvation gas flow 600 L/h, nitrogen nebuliser gas flow 7 bar; the multicharged ions signals were acquired in positive polarity mode in the range 50-2000 m/z using leucine enkephalin as mass reference compound; deconvoluted data were obtained using MaxEnt1 software (Waters).

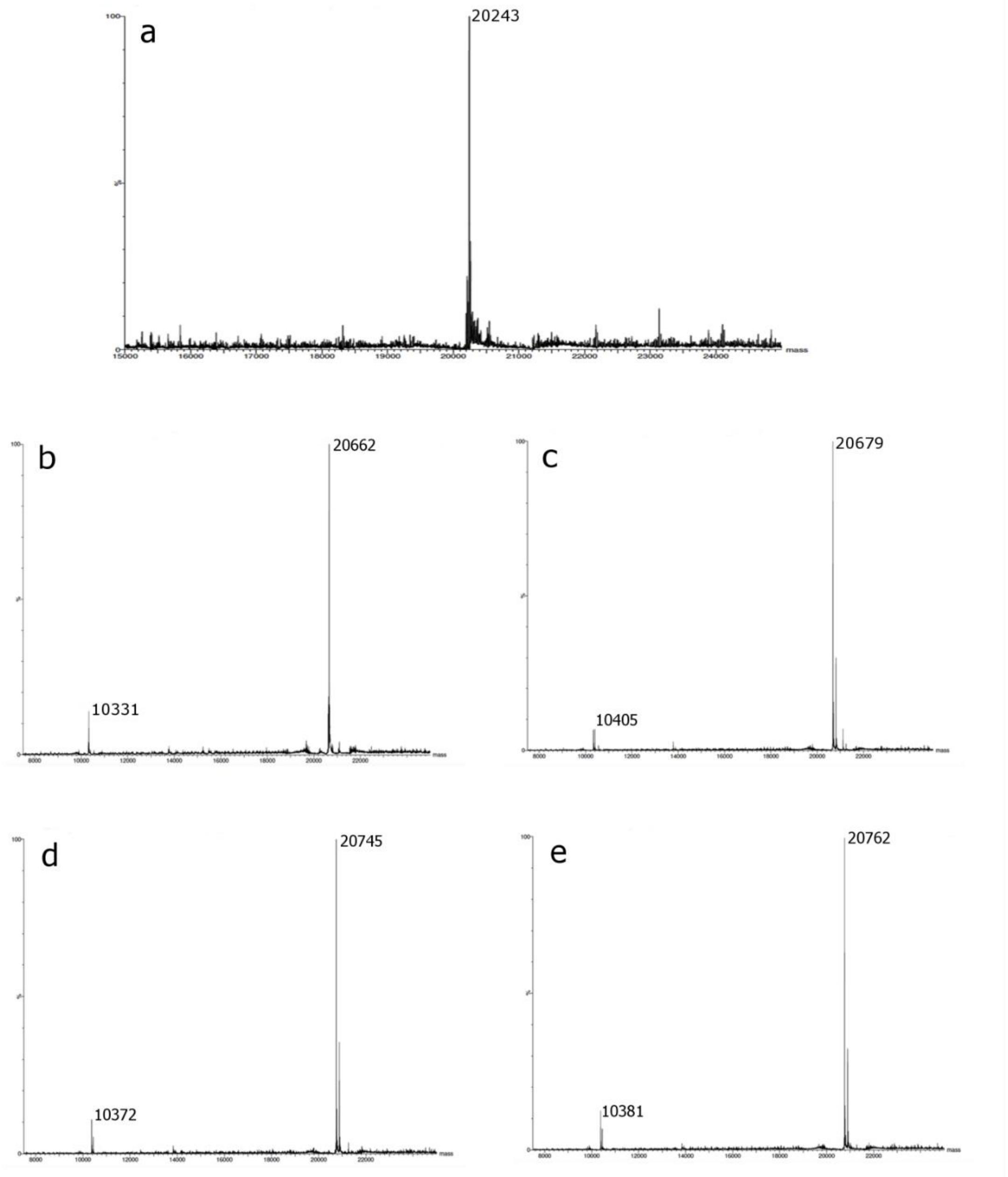

Figure S27: Assessment of the conjugation reactions: mass spectrometry analysis confirmed the expected molecular weight of a) HumAfFt (20243 Da) missing the first methionine and the expected molecular mass of the polyamines conjugated HumAfFt b) HumAfFt + **PA2.1** = 20662 Da. The m/z of 10331 corresponds to the doubly charged ion; c) HumAfFt + PA2.2 = 20679 Da. The m/z of 10405 corresponds to the doubly charged ion. d) HumAfFt + **PA 3.1** = 20745 Da. The m/z of 10372 corresponds to the doubly charged ion; e) HumAfFt + **PA3.2** = 20762 Da. The m/z of 10381 corresponds to the doubly charged ion.

5. Assessment of HumAfFt oligomerization

5.1 Size Exclusion Chromatography analysis

A solution of 1 mL of 2 mg mL^{-1} HumAfFt was reacted with each compound in 20 mM Hepes, 50 mM $MgCl_2$, pH 7.4 was injected on a HiLoad 16/600 Superdex 75 pg connected to an AKTA Pure (GE-Healthcare) pre-equilibrated in the same buffer. The retention time was compared with a sample of purified HumAfFt.

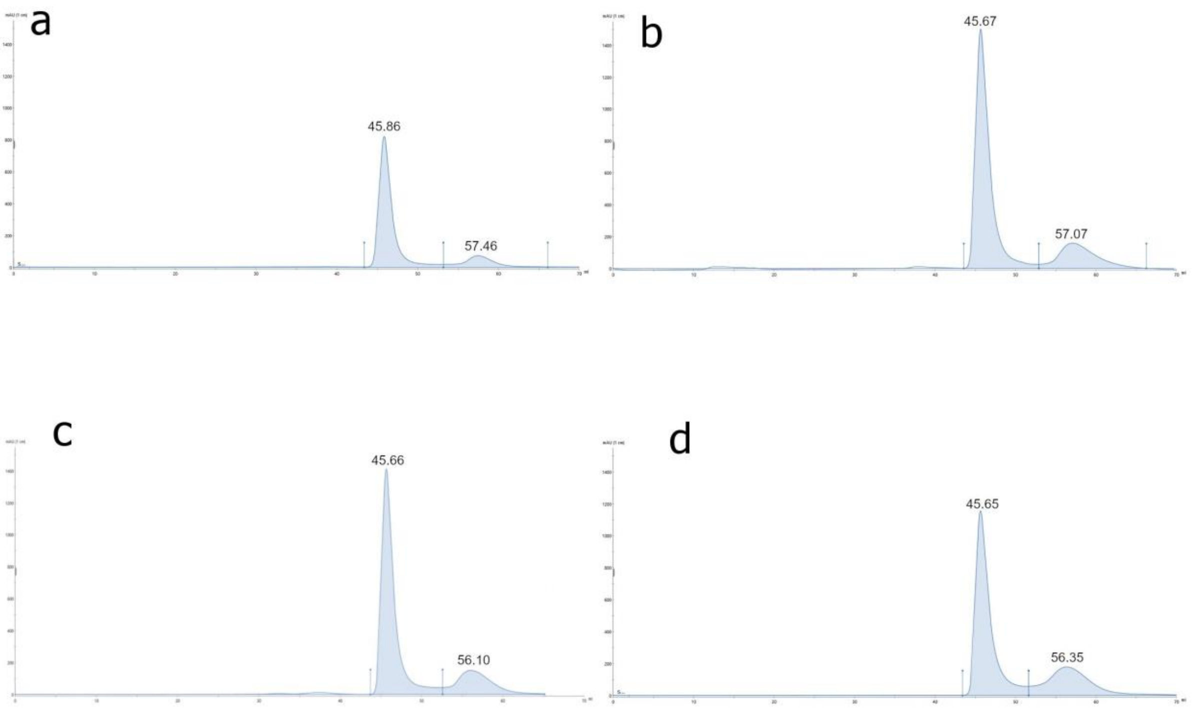

	24-mer Content (%)	Dimer Content (%)
HumAfFt + PA2.1	81	15
HumAfFt + PA2.2	76	18
HumAfFt + PA3.1	77	19
HumAfFt + PA3.2	69	25

Figure S28: Assessment of the HumAfFt oligomerization after conjugation reactions: SEC chromatograms showing the assembled 24-meric conformer peak at 45 min and the dimer at 56 min after conjugation with a) **PA2.1**, b) **PA2.2**, c) **PA3.1** and d) **PA3.2**. In the table, the content of the oligomerization state is reported for each compound.

5.2 Dynamic Light Scattering

Dynamic Light Scattering (DLS) experiments were carried out using a Zetasizer Nano S (Malvern Instruments, Malvern, UK) equipped with a 4 mW He–Ne laser (633 nm). Briefly, the measurements were performed at 25°C, at an angle of 173° with respect to the incident beam. The average hydrodynamic diameters (Z-average diameter) of the scattering particles were calculated using peak intensity analyses. Samples were prepared at 1 mg/mL in 20 mM Hepes, 50 mM $MgCl_2$, pH 7,4. All the traces for DLS experiments were analyzed with the software Origin 8.0 (Originlab Corporation, Northampton, MA, USA).

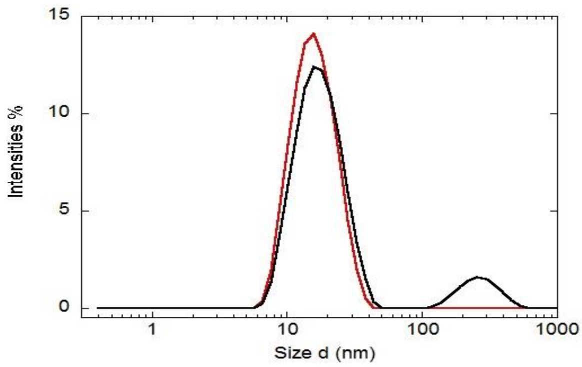

Figure S29: Dynamic light scattering profiles of Pas-HumAfFt before (red) and after encapsulation with siRNA (black).

6. siRNA sequences

All siRNAs and FITC-siRNAs targeting GAPDH were synthesized by Sigma Aldrich and purified by desalting chromatography. FITC-siRNAs were designed with a fluorescein group at 5’ in the sense sequence. The sequences are shown below:

siRNA Targeting GADPH	Sense sequence	Antisense sequence
	5'GGUUUACAUGUUCCAAUAU[dT][dT]	5'AUAUUGGAACAUGUAAACC[dT][dT]
	5'CUGACCUGCCGUCUAGAAA[dT][dT]	5'UUUCUAGACGGCAGGUCAG[dT][dT]
	5'GUCAACGGAUUUGGUCGUA[dT][dT]	5'UACGACCAAAUCCGUUGAC[dT][dT]
	5'CACAUGGCCUCCAAGGAGU[dT][dT]	5'ACUCCUUGGAGGCCAUGUG[dT][dT]

7. TfR1 expression by Immunoblotting

Cells were lysed and analyzed by electrophoresis as already described in the Experimental Section, immunoblotting was performed with the following antibodies: anti-CD71 (VMA00037) from BioRad; anti-GAPDH (MAB-10578) from Immunological Sciences.

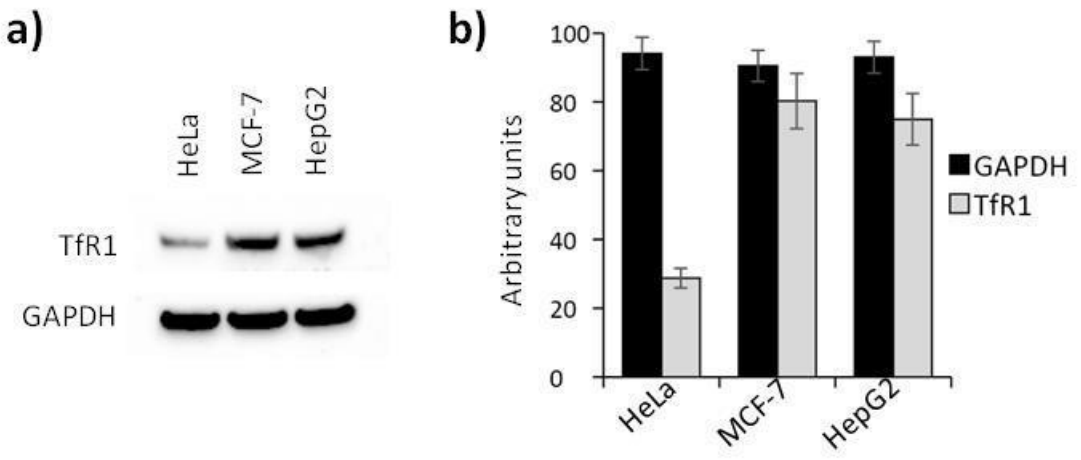

Figure S30: a) Total protein lysates were extracted from HeLa, MCF-7 and HepG2 and analyzed by immunoblotting using specific antibodies as indicated. b) Histogram showing immunoblot's densitometric analysis (ImageJ software), bars indicate SD.

8. FITC-siRNA-PAs-HumAfFt delivery by Flow Citometry

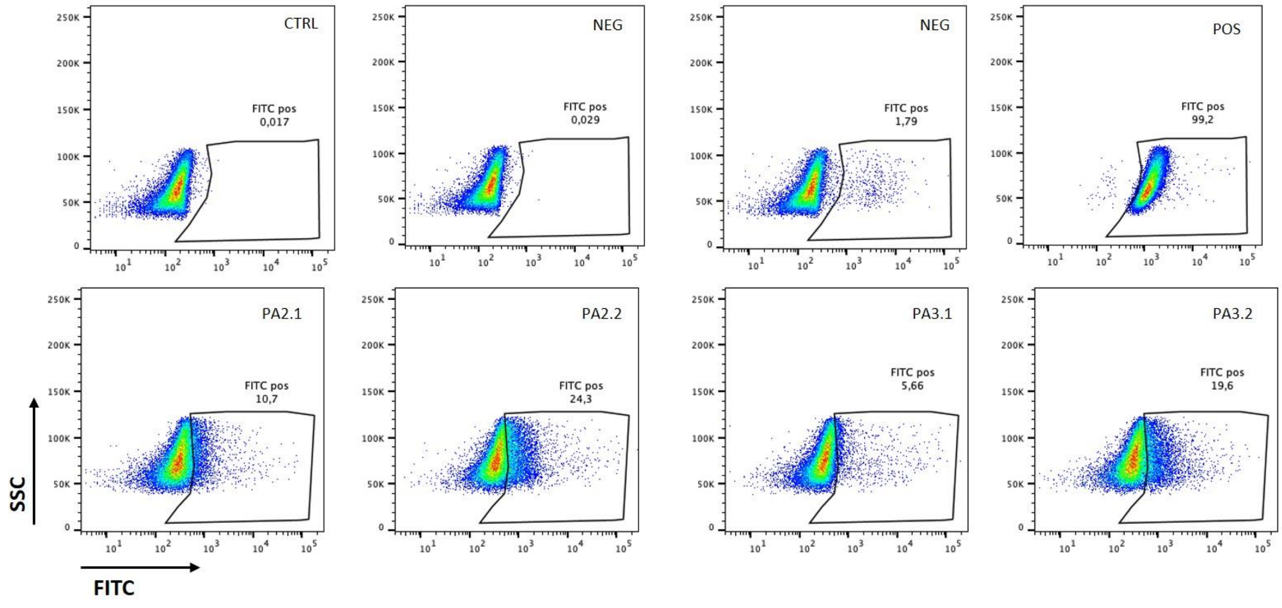

Figure S31: Flow Cytometry analysis of FITC-siRNA-PAs-HumAfFt delivery. Cells were treated and data were acquired as described in the Material and Methods section. As shown in the first line of the plot, the gate for the final detection was set in the control sample (CTRL), naked siRNA and FITC-siRNA show no detectable uptake (NEG) and FITC-plain-HumAfFt (POS) confirm a 99% of internalization under these experimental conditions. FACS analysis of HeLa cells confirm the uptake of FITC-siRNA-PAs-HumAfFt. In the second line, one of two independent experiments is shown as representative and the percentage of FITC positive cells for each sample is indicated in each plot. For each sample 30,000 events gated on live cells were acquired.

9. References

1. AXS, S. B.; Madison, W. J. S. U. G., Version, USA, 2004; SAINT, Software Users Guide, Version 6.0; Bruker Analytical X-ray Systems. **1999,** *6*.
2. A. Altomare; M. C. Burla; M. Camalli; G. L. Cascarano; C. Giacovazzo; A. Guagliardi; A. G. G. Moliterni; G. Polidori; R. Spagna, SIR99, a program for the automatic solution of small and large crystal structures *J. Appl. Crystallogr.* **1999,** *32*, 115-119.
3. Sheldrick, G. M. J. A. C. S. C. S. C., Crystal structure refinement with SHELXL. **2015,** *71* (1), 3-8.

10. Author contributions

Natalia Pediconi, Paola Baiocco and Alberto Boffi designed the biological experiments, analyzed the data and contributed to manuscript writing. Bruno Botta, Deborah Quaglio and Paola Baiocco conceived the project, provided overall guidance, and contributed to manuscript writing. Deborah Quaglio contributed to the rational design, development, and optimization of the synthetic approach. Paola Baiocco designed and purified HumAfFt, performed protein conjugations of polyamine-thiol-reactive linkers and experiments of siRNA encapsulation into the PAs-HumAfFt. Alessandro Arcovito performed light scattering experiments. Natalia Pediconi designed and performed the experiments on cancer cells and analysed the data. Giovanna Peruzzi designed and performed FACS experiments. Francesca Ghirga and Cristina Del Plato performed the synthesis, the acquisition and interpretation of Nuclear Magnetic Resonance spectroscopy (NMR) spectra of polyamine-thiol-reactive linkers. Constantinos Athanassopoulos contributed to the PAs synthetic part. Mattia Mori performed *ab initio* calculation of pK_a values of polyamine-thiol-reactive linkers. Maria Elisa Crestoni and Davide Corinti performed the acquisition and interpretation of the high-resolution mass spectra (HRMS) of polyamine-thiol-reactive linkers. Franco Ugozzoli and Chiara Massera performed crystallographic analysis.
